# A Frequência de Doenças Cardiovasculares na Artrite Reumatoide no Brasil: Estudo de Coorte de 10 Anos com Bancos de Dados do DATASUS

**DOI:** 10.36660/abc.20240313

**Published:** 2025-02-27

**Authors:** Roberto Gamarski, Fernando Ferreira de Castro, Jose Antonio Sena do Nascimento, Mirhelen Mendes de Abreu

**Affiliations:** 1 Universidade Federal do Rio de Janeiro Rio de Janeiro RJ Brasil Universidade Federal do Rio de Janeiro, Rio de Janeiro, RJ – Brasil

**Keywords:** Artrite Reumatoide, Doenças Cardiovasculares, Avaliação de Resultados em Cuidados de Saúde, Antirreumáticos

## Abstract

**Fundamento:**

Os desfechos cardiovasculares em pacientes com artrite reumatoide (AR) têm sido amplamente investigados na literatura em relação aos fatores biológicos.

**Objetivos:**

O presente estudo de coorte retrospectivo nacional visou explorar a distribuição de eventos cardiovasculares em pacientes com AR, assistidos pelo Sistema Único de Saúde (SUS) no Brasil, bem como identificar fatores associados a esses desfechos.

**Métodos:**

Pacientes com idade ≥ 18 anos foram identificados no Banco de Dados do Sistema Único de Saúde (DATASUS) por meio dos códigos CID-10 da AR e seus procedimentos terapêuticos de acordo com as diretrizes do SUS. Os pacientes com AR tratados com medicamentos antirreumáticos modificadores da doença (DMARDs) foram categorizados como usuários biológicos e não biológicos (sintéticos). Foram analisados os desfechos cardiovasculares, incluindo doença arterial coronária aguda (DACA), insuficiência cardíaca e acidente vascular cerebral (AVC). Os pacientes também foram categorizados com base nos padrões de tratamento (se usaram o medicamento consistentemente ou mudaram para outro). O nível socioeconômico foi avaliado usando o Índice FIRJAN de Desenvolvimento Municipal (IFDM). As análises descritivas identificaram a distribuição populacional e os desfechos cardiovasculares, e a regressão logística múltipla explorou os fatores associados. A significância estatística adotada foi de p < 0,05.

**Resultados:**

Entre os 4.321 pacientes com AR tratados com DMARDs, foram identificados 198 desfechos cardiovasculares (4,68%). A maioria era do sexo feminino (3.398 [80,3%]) com idade média de 54,2 (desvio padrão 12,8) anos, predominantemente da Região Sudeste (2.421 [57,2%]). O IFDM geral predominante foi > 0,8 (47,5%). Idade avançada, presença de fatores de risco cardiovascular e uso de DMARDs sintéticos foram associados aos desfechos cardiovasculares.

**Conclusão:**

Os desfechos cardiovasculares em pacientes com AR são comuns e estão associados à idade, comorbidades e medicamentos usados para tratamento.

## Graphical abstract

**Figura Central:**
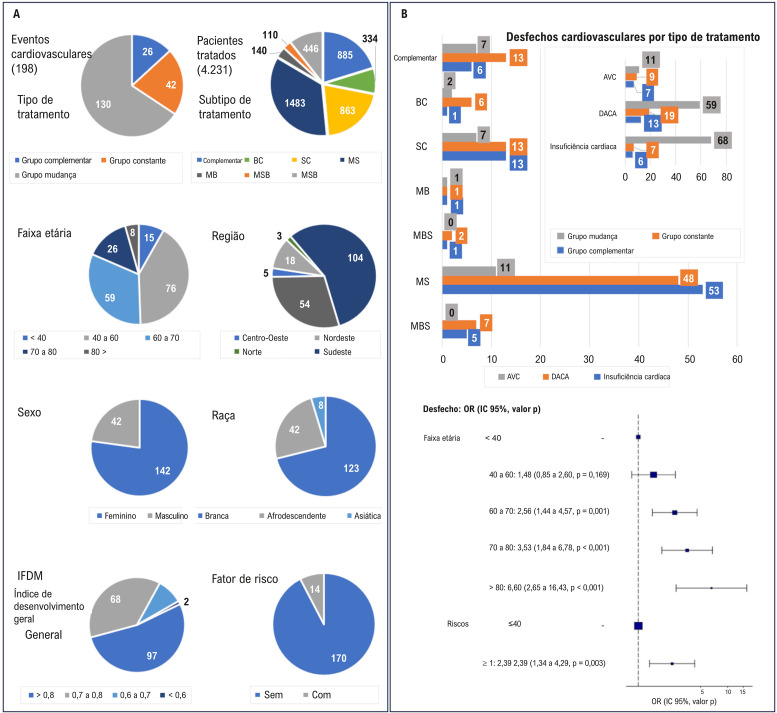
: A Frequência de Doenças Cardiovasculares na Artrite Reumatoide no Brasil: Estudo de Coorte de 10 Anos com Bancos de Dados do DATASUS

AVC: acidente vascular cerebral; BC: biológico constante; DACA: doença arterial coronária aguda; MB: mudança entre biológicos; MBS: mudança de biológico para sintético; IC: intervalo de confiança; IFDM: Índice FIRJAN de Desenvolvimento Municipal; MS: mudança entre sintéticos; MSB: mudança de sintético para biológico; OR: razão de chances; SC: sintético constante.

## Introdução

Eventos cardiovasculares são uma preocupação significativa na etiopatogenia e história natural da artrite reumatoide (AR).^1^ Marcadores inflamatórios elevados em pacientes com AR estão associados ao aumento da mortalidade cardiovascular e à maior atividade da doença.^2 , 3^ Embora fatores de risco cardiovascular tradicionais, como hipertensão e diabetes, sejam bem conhecidos, também foi destacado na literatura o papel dos tratamentos para AR na redução dos riscos cardiovasculares, particularmente os medicamentos antirreumáticos modificadores da doença (DMARDs, do inglês *disease-modifying antirheumatic drugs* ) biológicos.^4^

O presente estudo utiliza dados do DATASUS, o sistema de informações de saúde do Sistema Único de Saúde (SUS) do Ministério da Saúde do Brasil,^5^ que cobre 80% da população brasileira. Ao explorar dados desse sistema abrangente, nosso estudo visa descrever a distribuição de doenças cardiovasculares em pacientes com AR tratados com DMARDs no Brasil e identificar os fatores associados a esses resultados. Os DMARDs avaliados são descritos na Metodologia.

Considerando que indicadores socioambientais como condições climáticas, poluição e índice de desenvolvimento humano têm sido menos explorados,^6 , 7^ o estudo avaliou o Índice FIRJAN de Desenvolvimento Municipal (IFDM), um indicador sintético de qualidade de vida municipal, que é detalhado na Metodologia e no Material Suplementar Online. Especificamente, visamos entender a interação entre DMARDs sintéticos e biológicos na prevenção de desfechos cardiovasculares (Material Suplementar Online, Seção 1), descrever a distribuição de doenças cardiovasculares nesse grupo de pacientes e identificar o papel dos fatores que contribuem para os desfechos, como comorbidades, fatores demográficos e socioeconômicos. O presente estudo busca preencher lacunas no conhecimento atual e fornecer insights sobre como esses tratamentos impactam a saúde cardiovascular em pacientes com AR no contexto brasileiro.

O problema principal a ser abordado é entender a frequência dos desfechos cardiovasculares em pacientes com AR. Alguns fatores afetam esses desfechos e são chamados de multifatoriais na literatura; portanto, buscamos identificar a relevância desses fatores, tais como variáveis demográficas, sociais e clínicas. Os resultados do presente projeto podem contribuir para o desenvolvimento do conhecimento científico sobre a interação entre doença cardiovascular e AR, identificando o papel dessas variáveis multifatoriais.

## Métodos

Os procedimentos metodológicos iniciaram com a integração de bases de dados administrativos do DATASUS, que ocorreu nas seguintes três fases: (1) construção e validação dos algoritmos utilizados para identificar os sujeitos na base de dados previamente extraída, (2) construção e validação dos algoritmos utilizados para identificar variáveis demográficas e clínicas, (3) aplicação dos algoritmos e teste do modelo para analisar a associação entre essas variáveis e desfechos cardiovasculares.

### Delineamento

O presente estudo foi uma coorte retrospectiva longitudinal observacional com extração de dados nacionais do DATASUS entre 2008 e 2018 ( Material Suplementar Online, Seção 2 ). Os pacientes foram incluídos no estudo de 2009 a 2017, e o período de estudo para cada paciente foi de 4 anos (48 meses), com acompanhamento censurado devido a morte, perda de acompanhamento ou conclusão do período de estudo.^8^

### Extração de dados

A extração de dados foi realizada individualmente e de forma anônima.^9^ A definição de AR para a busca nos sistemas de dados administrativos foi orientada pela construção de algoritmos ( Material Suplementar Online, Seção 3 ) em alinhamento com os Códigos Internacionais de Doenças (CID-10) para AR e suas subclassificações (CID-10 de M05 a M08), em associação com os códigos de procedimentos dos Protocolos Clínicos e Diretrizes Terapêuticas (PCDT) para AR autorizados e cobertos pelo SUS.^10^

Os dados foram integrados de três sistemas de dados administrativos do DATASUS: Autorização de Procedimento de Alto Custo (APAC), Boletim de Produção Ambulatorial Individualizado (BPAi) e Autorização de Internação Hospitalar (AIH). Detalhes desses sistemas podem ser verificados no Material Suplementar Online ( Figura S1 ). O sistema APAC inclui informações sobre data de nascimento, sexo, estado, cidade, código de doença reumática do CID, fase do tratamento (inicial ou retorno), tratamento imunossupressor, diálise (sim ou não) e consultas profissionais durante o período do estudo. O AIH inclui as datas de internação, alta e nascimento, sexo, cidade, tempo de internação, CID do motivo para internação, data e causa do óbito quando aplicável, tipo de internação (urgência ou emergência) e motivo da desospitalização (cura ou óbito). O BPAi inclui dados do paciente como cartão nacional de saúde, sexo, data de nascimento, raça, cor e etnia, além do código do município de residência. Inclui também o Cadastro Nacional de Estabelecimentos de Saúde (CNES) do estabelecimento realizador, código do procedimento, quantidade de procedimentos realizados, data e CID respectivo. O escopo da extração foi nacional, realizado entre 2008 e 2018.

### Critérios de elegibilidade e definição de casos

**Figura 1:**
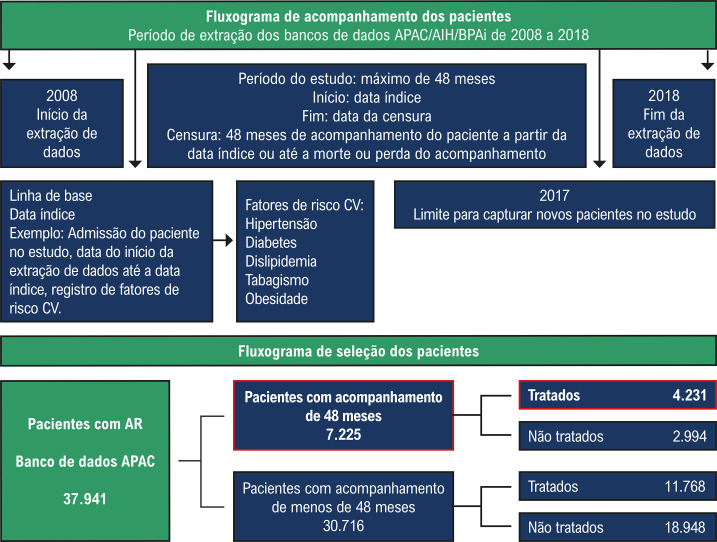
– Fluxograma de acompanhamento e seleção de pacientes. Notas: O fluxograma de acompanhamento dos pacientes mostra os procedimentos para identificação do início ao fim da extração de dados. O fluxograma de seleção dos pacientes mostra a seleção dos pacientes incluídos no estudo, destacando os pacientes com acompanhamento de 48 meses e em tratamento com medicamentos antirreumáticos modificadores da doença (n = 4.231). AIH: Autorização de Internação Hospitalar; APAC: Autorização de Procedimento de Alto Custo pelo Sistema Único de Saúde; AR: artrite reumatoide; BPAi: Boletim de Produção Ambulatorial Individualizado; CV: cardiovascular Fonte: elaboração dos autores.

Os pacientes com AR eram selecionados se tivessem mais de 18 anos, conforme descrito pela data de nascimento nos formulários do DATASUS usados no estudo. Os pacientes tinham que ter informações contínuas em pelo menos um dos bancos de dados por até 6 meses consecutivos antes da data da linha de base. Pacientes com mais de 20% de informações ausentes em pelo menos duas variáveis do estudo foram excluídos ( Figura 1 ). O fluxograma de seleção de pacientes e o número de pacientes incluídos no estudo são fornecidos na Figura 1 .

### Limpeza de dados e construção de variáveis

Após a extração inicial de dados, foi realizado um processo de limpeza completo para garantir a qualidade e a consistência dos dados. Isso incluiu a remoção de entradas duplicadas, resolução de inconsistências e tratamento de dados ausentes. As variáveis foram construídas com base nos dados disponíveis, garantindo que refletissem com precisão as características demográficas, clínicas e socioeconômicas dos pacientes.

Inicialmente, foi realizada uma análise exploratória da distribuição de medicamentos descritos nos sistemas de dados. As informações sobre o tratamento são obtidas especificamente no banco de dados do APAC, que concentra informações sobre o uso de medicamentos de alto custo. Com base nessa distribuição e nas suposições descritas, os pacientes com AR foram categorizados nos seguintes grupos: complementar, constante ou mudança. Pacientes no grupo complementar são aqueles que adicionaram qualquer medicamento específico para AR durante o acompanhamento. Pacientes constantes são aqueles que não alteraram o regime terapêutico durante o acompanhamento. Pacientes no grupo mudança são aqueles que alteraram o regime terapêutico durante o acompanhamento. O grupo constante foi subclassificado como sintético ou biológico. O grupo mudança foi subclassificado de acordo com a mudança entre DMARD biológico para DMARD sintético ou vice-versa, ou mesmo quando houve uma mudança entre medicamentos da mesma categorização, ou seja, de um medicamento biológico para outro ou de um medicamento sintético para outro ( Material Suplementar Online, Tabela S1 ). Os seguintes DMARDs foram avaliados no grupo sintético: metotrexato, leflunomida, cloroquina ou hidroxicloroquina, sulfassalazina. O grupo biológico incluiu os seguintes DMARDs: adalimumabe, infliximabe, etanercepte, certolizumabe, golimumabe, tocilizumabe, abatacepte e rituximabe.

### Perfil das comorbidades clínicas

Para o presente estudo, a gravidade da AR foi categorizada de acordo com o tipo de tratamento específico para a doença recebido durante o acompanhamento. As informações sobre o tratamento contidas nos sistemas de dados foram consideradas ( Material Suplementar Online, Tabela S2 ) com base nos seguintes princípios: (1) em consonância com as diretrizes brasileiras utilizadas para o presente estudo, o medicamento biológico é fornecido como tratamento de segunda linha, e sua introdução sugere falha do regime de primeira linha;^10^ (2) as mudanças no tratamento refletem uma mudança no estado de saúde, ou seja, qualquer adição ou mudança de tratamento refletiria piora clínica, enquanto a remoção de um determinado medicamento do sistema de informação foi considerada como proxy para melhora no estado de saúde do paciente com AR.^11^

As informações socioeconômicas relacionadas ao local de residência também foram obtidas e analisadas, sendo a menor unidade geográfica disponível para análise o município e o distrito de saúde onde a doença foi tratada. A presente pesquisa foi baseada em informações do IFDM, um indicador sintético da qualidade de vida municipal ( Material Suplementar Web, Seção 4 ). O IFDM monitora anualmente o desenvolvimento socioeconômico de todos os municípios brasileiros em 3 áreas de atuação: emprego/renda, educação e saúde.^12^ Esse indicador foi construído com base unicamente em estatísticas públicas oficiais disponibilizadas pelos Ministérios do Trabalho, Educação e Saúde do Brasil, e pode ser lido em aspectos gerais ou analisado por setor em termos de emprego/renda, saúde e educação. O índice varia de 0 (mínimo) a 1 ponto (máximo) para classificar o nível de cada localidade nas seguintes 4 categorias: baixo (0 a 0,4), regular (0,4 a 0,6), moderado (0,6 a 0,8) e alto (0,8 a 1) ( Material Suplementar Online, Tabela S3 ).

### Fatores de risco cardiovascular

Os fatores de risco cardiovascular foram identificados a partir de todos os sistemas de dados administrativos do DATASUS com base na presença do conjunto de informações agrupadas pelo CID-10 da taxa de morbidade em associação com os códigos de procedimentos para seu atendimento. Os eventos cardiovasculares foram definidos como DACA, insuficiência cardíaca e AVC. Os eventos cardiovasculares foram categorizados por faixa etária (> 40, 40 a 60, 60 a 70, 70 a 80, > 80 anos). O perfil de comorbidades clínicas foi definido com foco no mapeamento de fatores de risco cardiovascular tradicionais, incluindo dislipidemia, hipertensão, diabetes, obesidade e tabagismo.

### Análise estatística

A distribuição dos pacientes na linha de base foi descrita a partir de dados demográficos, dados de AR (categorizados por tratamento), fatores de risco tradicionais para eventos cardiovasculares e fatores socioeconômicos (IFDM). As variáveis categóricas foram descritas usando frequências absolutas e relativas. Após esta etapa, foi realizada a análise descritiva dos desfechos cardiovasculares. Subsequentemente, foi usado um modelo de regressão logística para explorar os fatores de linha de base associados à ocorrência de desfechos cardiovasculares na população estudada. As etapas para a construção do modelo foram as seguintes:

1. Emparelhamento da amostra com base na presença de desfechos cardiovasculares usando variáveis como sexo, faixa etária, região, diabetes, obesidade, hipertensão, tabagismo e dislipidemia, com uma proporção de pareamento de 1:5.
2. Teste qui-quadrado para verificar diferenças entre modelos de tratamento dada a presença do desfecho. ^13^
3. Teste *post-hoc* pareado para comparar proporções e identificar diferenças entre modelos quando o teste qui-quadrado foi significativo.
4. Método de Bonferroni para corroborar os resultados do valor p (a significância estatística adotada foi de p < 0,05). ^14^
5. Cálculo de razão de chances para revelar resultados estatisticamente significativos.

A análise coloca o uso das ferramentas estatísticas em uma ordem apropriada, com o teste qui-quadrado aplicado inicialmente para detectar diferenças entre modelos de tratamento com base na presença de desfechos, o que é adequado para amostras independentes. Em segundo lugar, o modelo *stepwise* foi usado para selecionar quais variáveis seriam associadas aos desfechos. Nesse processo, excluímos primeiramente a variável modelo de tratamento. Em sequência, as variáveis foram selecionadas pelo modelo *stepwise* como critérios no pareamento entre pacientes com desfechos e pacientes sem desfechos. Com os pacientes pareados, usamos o teste de proporções para verificar em qual subgrupo dentro do modelo de tratamento havia significância estatística. Isso foi feito para ter certeza de que o efeito de outras variáveis seria anulado e para excluir o efeito de confusão na magnitude do efeito do tratamento no grupo de pacientes com desfechos. Após verificar em qual grupo houve diferença, calculamos a razão de chances correspondente e valor de p, considerando p < 0,001 ( Material Suplementar Online, Tabela S8 ). Essa abordagem nos permitiu controlar as variáveis de confusão e conduzir uma análise precisa dos subgrupos.

O estudo foi submetido e aprovado pelo Comitê de Ética em Pesquisa Institucional, sob o parecer número 23016819.9.0000.5257. A extração dos dados foi fornecida pela gerência do DATASUS por meio processo número SEI 250001348282015-86.

## Resultados

### População do estudo

Inicialmente, foram identificados 37.941 pacientes com diagnóstico de AR, dos quais 7.225 foram acompanhados por um período de 48 meses, e 4.231 deles foram tratados com DMARDs. A análise do estudo, portanto, incluiu 4.231 pacientes com AR tratados com DMARDs e acompanhados por 48 meses ( Figura 1 ). A maioria dos pacientes era do sexo feminino (80,3%), com média de idade de 54,2 anos. A maioria dos pacientes residia na Região Sudeste (57,2%) e 47,5% tinham IFDM acima de 0,8. A Tabela 1 apresenta a distribuição social, demográfica e clínica da população tratada em relação à linha de base e aos 48 meses. A Ilustração Central fornece uma visão geral da distribuição social, demográfica e clínica da população com AR e desfechos cardiovasculares.

**Tabela 1:** – Distribuição social, demográfica e clínica da população tratada (linha de base e 48 meses)

Variável	Linha de base (n = 15.999)	48 meses (n = 4.231)	Observações
Sexo			
Feminino	12.779 (79,9%)	3.398 (80,3%)	Pacientes predominantemente do sexo feminino.
Masculino	3.220 (20,1%)	833 (19,7%)	
Idade			
Média	50,08 (13,82)	54,22 (12,83)	Aumento da idade média ao longo dos 48 meses.
< 40 anos	3.888 (24,3%)	648 (15,3%)	Diminuição de pacientes mais jovens.
40 a 60 anos	8.480 (53,0%)	2.210 (52,2%)	Proporção consistente de pacientes de meia-idade.
60 a 70 anos	2.645 (16,5%)	993 (23,5%)	Aumento no número de pacientes mais velhos.
70 a 80 anos	862 (5,4%)	324 (7,7%)	
> 80 anos	124 (0,8%)	56 (1,3%)	
Raça			
Branca	7.559 (47,2%)	2.138 (50,5%)	Pacientes predominantemente brancos.
Afrodescendente	3.440 (21,5%)	834 (19,7%)	
Asiática	312 (2,0%)	84 (2,0%)	
Região			
Centro-Oeste	898 (5,6%)	195 (4,6%)	
Norte	551 (3,4%)	91 (2,2%)	
Nordeste	2.332 (14,6%)	474 (11,2%)	
Sul	3.268 (20,4%)	1.050 (24,8%)	
Sudeste	8.950 (55,9%)	2.421 (57,2%)	Predominantemente da Região Sudeste.
IFDM			
> 0,8	7.086 (44,3%)	2.010 (47,5%)	Maioria com alto IFDM.
IFDM emprego/renda			
0,6 a 0,7	7.036 (44,0%)	1.756 (41,5%)	
IFDM saúde			
> 0,8	12.492 (78,1%)	3.443 (81,4%)	Maioria com alto IFDM saúde.
IFDM educação			
> 0,8	11.251 (70,3%)	3.031 (71,6%)	Maioria com alto IFDM educação.
Fatores de risco CV			
0	15.485 (96,8%)	4.102 (97,0%)	Maioria sem fatores de risco (p = 0,003).
≥ 1	514 (3,2%)	129 (3,0%)	

A tabela mostra um perfil demográfico e clínico abrangente dos pacientes com artrite reumatoide na linha de base e após 48 meses. Há um aumento notável na idade média e na proporção de pacientes mais velhos ao longo do período do estudo. A maioria dos pacientes é da Região Sudeste e tem alto IFDM nos setores de saúde e educação. CV: cardiovascular; IFDM: Índice FIRJAN de Desenvolvimento Municipal. Fonte: elaboração dos autores.

### Distribuição e categorização dos DMARDs

Os DMARDs biológicos foram usados por 1.885 (44,6%) pacientes, enquanto DMARDs sintéticos foram usados por 3.757 (88,8%) pacientes. Os pacientes foram categorizados com base em suas trajetórias de tratamento da seguinte forma: sintético constante (20,4%), mudança entre sintéticos (35,1%), biológico constante (7,9%), medicamento complementar (20,2%), mudança de sintético para biológico (10,5%), mudança entre biológicos (3,3%) e mudança de biológico para sintético (2,6%). O grupo mudança entre sintéticos mostrou uma prevalência maior de fatores de risco cardiovascular tradicionais ( Material Suplementar Online, Tabela S4 ).

### Desfechos cardiovasculares

**Figura 2:**
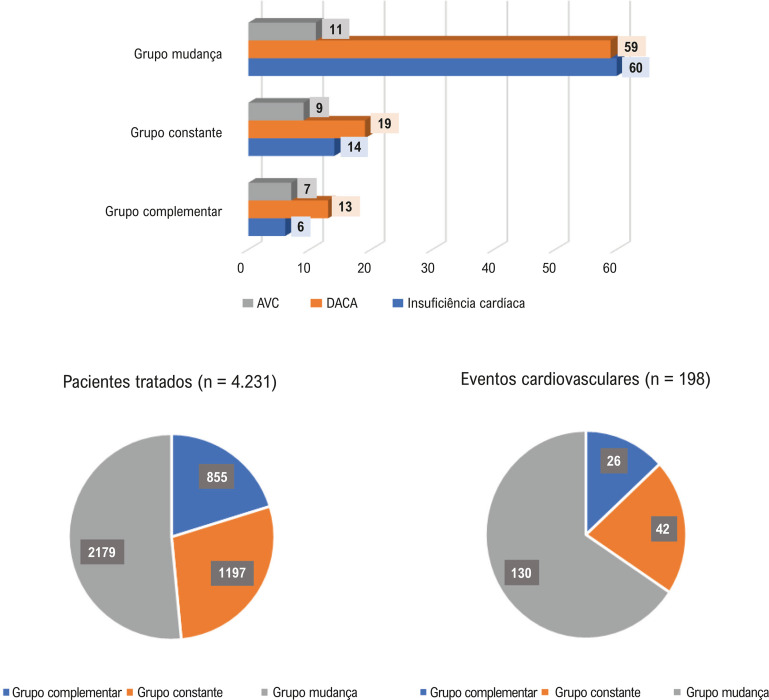
– Distribuição dos desfechos cardiovasculares em relação às categorias agrupadas de tratamento. Descrição: Gráfico de barras mostrando a distribuição dos desfechos cardiovasculares por categorias de tratamento. Complementar: adição de um novo medicamento biológico ou sintético; constante: o mesmo medicamento durante o estudo; mudança: troca de um medicamento para outro durante o estudo. AVC: acidente vascular cerebral; DACA: doença arterial coronária aguda. Fonte: elaboração dos autores.

Durante o período de acompanhamento, 198 desfechos cardiovasculares foram registrados em 184 pacientes, incluindo insuficiência cardíaca, síndromes coronárias agudas e AVC. Conforme ilustrado nas Figuras 2 e 3 , a maioria dos resultados ocorreu no grupo mudança (130 [65,7%]), seguido pelo grupo constante (42 [21,2%]) e pelo grupo complementar (26 [13,1%]). Os desfechos foram mais frequentes no grupo mudança entre sintéticos (112 [56,6%]), seguido pelo grupo sintético constante (33 [16,6%]), pelo grupo complementar (26 [13,3%]), pelo grupo mudança de sintético para biológico (12 [6%]), pelo grupo biológico constante (9 [4,5%]), pelo grupo mudança entre biológicos (3 [1,5%]) e pelo grupo mudança de biológico para sintético (3 [1,5%]) ( Material Suplementar Online, Tabela S5 ).

### Características dos pacientes com desfechos cardiovasculares

**Figura 3:**
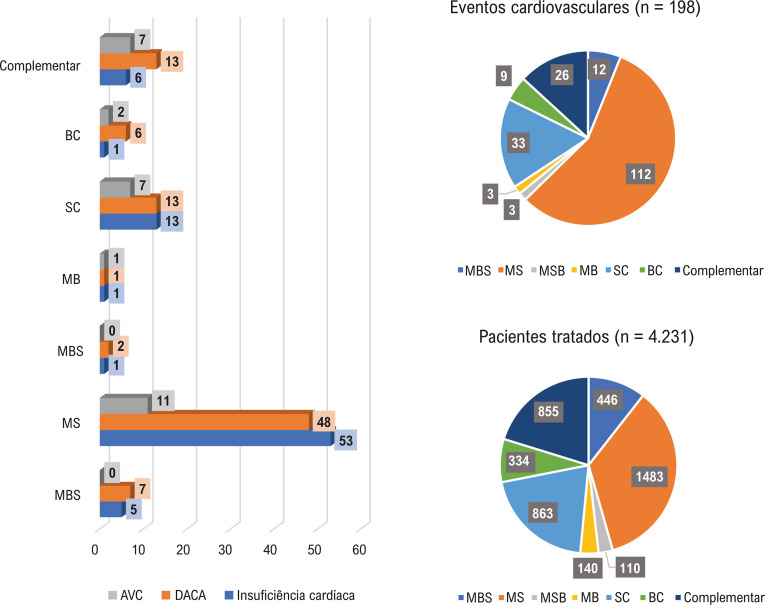
– Distribuição de desfechos cardiovasculares em relação a cada categoria de tratamento para artrite reumatoide. Descrição: Gráfico detalhado mostrando a distribuição de desfechos cardiovasculares por subcategorias específicas de tratamento. Complementar: adição de um novo medicamento biológico ou sintético; BC: biológico constante; AVC: acidente vascular cerebral; DACA: doença arterial coronária aguda; MB: mudança entre biológicos; MBS: mudança de biológico para sintético; MS: mudança entre sintéticos; MSB: mudança de sintético para biológico; SC: sintético constante. Fonte: elaboração dos autores.

A distribuição dos desfechos cardiovasculares ao final do período de acompanhamento de 48 meses foi avaliada em relação ao total de 198 desfechos cardiovasculares. Os desfechos cardiovasculares identificados foram: DACA (CID-10 I20-I24) com 91 eventos (46%), insuficiência cardíaca (CID-10 I50) com 80 eventos (40,4%) e AVC (CID-10 I64/G45) com 27 eventos (13,6%) ( Figura 3 ). Dos 198 desfechos cardiovasculares, 77,2% ocorreram em mulheres; 41,3% dos pacientes tinham entre 40 e 60 anos, com média de idade de 59,93 anos. A maioria dos pacientes era branca (66,8%) e residia na Região Sudeste (56,5%). A maioria apresentou IFDM geral maior que 0,8 (52,7%), particularmente nos domínios de saúde (83,2%) e educação (71,7%), enquanto o domínio de renda/emprego variou de 0,6 a 0,7 (40,8%). Observamos que o domínio renda/emprego do IFDM foi predominantemente entre 0,6 e 0,7 (40,8%) no grupo com desfechos cardiovasculares, bem como para o grupo geral (41,5%), embora valha a pena notar que o IFDM foi analisado em todas as regiões brasileiras, e nenhuma significância estatística foi observada (p > 0,05) ( Material Suplementar Online, Tabela S6 ).

### Associação com fatores de risco cardiovascular tradicionais e desfechos cardiovasculares

**Figura 4:**
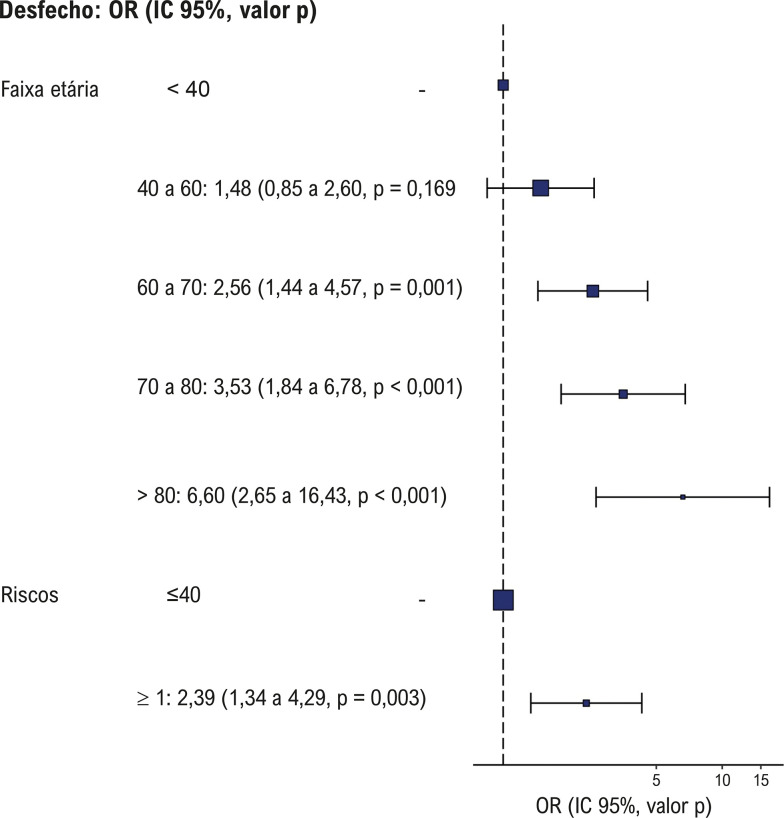
– Modelo de regressão logística para desfechos cardiovasculares. Descrição: Representação gráfica do modelo de regressão logística mostrando a razão de chances para eventos cardiovasculares. OR: razão de chances para eventos cardiovasculares. Fonte: elaboração dos autores.

Os desfechos cardiovasculares foram mais comuns em pacientes com fatores de risco tradicionais, com uma associação direta entre a presença de um ou mais fatores de risco e desfechos cardiovasculares (razão de chances de 2,39; p = 0,003) ( Figura 4 ). Houve uma relação crescente entre idade e ocorrência de desfechos cardiovasculares, com maior razão de chance para faixas etárias mais velhas. Pacientes no grupo mudança entre sintéticos tiveram maior probabilidade de desfechos cardiovasculares em comparação ao grupo sintético constante (razão de chances de 2,31; p = 0,002) ( Tabela 3 ; Material Suplementar Online, Tabela 7A e 7B ).

**Tabela 3:** – Distribuição dos DMARDs utilizados no estudo REAL e no presente estudo, apontando os desfechos cardiovasculares no presente estudo, de acordo com os DMARDs que os pacientes receberam

	Tipo de DMARDs	Estudo REAL e DMARDS	Presente estudo e DMARDs	Desfechos cardiovasculares
Pacientes	-	N = 1.116	N = 4.231	N = 184
Metotrexato	Sintético	66,5%	31,9%	29,9%
Leflunomida	Sintético	33,9%	59,6%	60,9%
Cloroquina ou hidroxicloroquina	Sintético	13,1%	18,4%	17,4%
Sulfassalazina	Sintético	4,9%	11,3%	15,2%
Anti-TNF (adalimumabe, infliximabe, etanercepte, certolizumabe, golimumabe,)	Biológico	19,9%	53,4%	31,0%
Tocilizumabe	Biológico	4,9%	1,7%	0
Abatacepte	Biológico	6,5%	2,0%	0
Rituximabe	Biológico	4,4%	1,5%	1,1%
Grupo sintético	-	-	3.757 (88,8%)	172 (93,5%)
Grupo biológico	-	-	1.885 (44,6%)	51 (27,7%)

A tabela especifica a associação de desfechos cardiovasculares e DMARDs sintéticos e biológicos usados no estudo REAL e em nosso estudo. DMARDs: medicamentos antirreumáticos modificadores da doença. Fonte: elaboração dos autores.

## Discussão

O aumento do risco de eventos cardiovasculares na AR não pode ser totalmente explicado apenas pelos fatores de risco tradicionais.^2^ O presente estudo de coorte retrospectivo visou identificar fatores de risco tradicionais e não tradicionais associados a desfechos cardiovasculares em pacientes com AR registrados no DATASUS ( Material Suplementar Online, Tabela S9 ).

### Achados sobre eventos cardiovasculares

Identificamos 198 eventos cardiovasculares em nossa coorte. O envelhecimento foi um fator prevalente entre pacientes com eventos cardiovasculares, sendo a hipertensão o fator de risco tradicional mais comum associado a esses eventos. Embora a proporção de fatores de risco tradicionais encontrados em nosso estudo tenha sido relativamente baixa em comparação a outros estudos, nossos achados se alinham com outros estudos de coleta de dados primários realizados no Brasil. Por exemplo, a prevalência relatada de hipertensão em pacientes com AR varia significativamente de 4% a 73%, dependendo do desenho do estudo e da população.^15 , 16^ Simard et al.^17^ mostraram que a prevalência de diabetes na AR está entre 7,0% e 35,0%, com poucos estudos indicando uma prevalência maior de diabetes na AR^18^ ( Material Suplementar Online, Tabela S10 ).

### Impacto dos DMARDs no risco cardiovascular

Vicente et al.^19^ realizaram uma análise transversal de pacientes da coorte do estudo REAL na linha de base, verificando que o risco cardiovascular foi alto na população com AR. O estudo sugeriu que a escolha de DMARDs, particularmente os biológicos, poderia resultar em melhores desfechos cardiovasculares em comparação aos sintéticos ( Tabela 3 ). Nosso estudo de coorte corroborou esses achados, mostrando uma associação entre o tipo de tratamento para AR e a ocorrência de desfechos cardiovasculares.^20^ Pacientes que precisaram mudar sua medicação para controlar a atividade da doença (grupo mudança) tiveram maior probabilidade de desenvolver desfechos cardiovasculares. Golmia et al.^21^ apresentaram uma comparação direta do uso de infliximabe e tocilizumabe, dois DMARDs biológicos, e verificaram diferentes respostas entre eles em relação à funcionalidade da AR.

### Modelo estatístico e associações principais

O modelo de regressão logística do nosso estudo verificou que a progressão da idade e a presença de um ou mais fatores de risco cardiovascular estavam significativamente associados aos desfechos cardiovasculares. O modelo mostrou uma associação direta entre a presença de fatores de risco cardiovascular tradicionais e desfechos cardiovasculares, com razão de chances de 2,39 (1,34 a 4,29; p = 0,003) ( Tabela 2A ; Figura 4 ). Em nossa análise pareada, observamos uma diferença significativa nos desfechos cardiovasculares entre pacientes que precisaram mudar entre sintéticos, sugerindo que esses pacientes podem ter doença mais grave ( Tabela S8 ).

**Tabela 2:** – Modelo de regressão para diferentes variáveis e associação com desfechos cardiovasculares e resultado do teste comparando as proporções entre os tipos de tratamento

Variável	OR (IC 95%)	Valor p (Teste de Wald)	Observações
Tabela 2A Idade (referência: < 40 anos)			
40 a 60 anos	1,48 (0,85 - 2,60)	0,169	Nenhuma associação significativa
60 a 70 anos	2,56 (1,44 - 4,57)	0,001	Associação significativa
70 a 80 anos	3,53 (1,84 - 6,78)	<0,001	Associação significativa
> 80 anos	6,60 (2,65 - 16,43)	<0,001	Associação significativa
Fator de risco (referência: 0)			
≥ 1 versus 0	2,39 (1,34 - 4,29)	0,003	Associação significativa
**Comparação**	**OR (95% CI)**	**Valor p**	**Observações**
Tabela 2B			
Mudança versus constante	1,81 (1,23 - 2,71)	0,056	Quase significativa
Tabela 2C			
Mudança de sintéticos versus sintético constante	2,31 (1,50 - 3,66)	0,002	Associação significativa
Mudança de sintéticos versus complementar	1,81 (1,15 - 2,96)	0,16	Nenhuma associação significativa

A Tabela 2A destaca as chances crescentes de desfechos cardiovasculares com o avanço da idade. A presença de um ou mais fatores de risco aumenta significativamente a probabilidade de eventos cardiovasculares. As Tabelas 2B e 2C mostram uma associação significativa entre a mudança de sintéticos e os desfechos cardiovasculares em comparação ao tratamento sintético constante. IC: intervalo de confiança; OR: razão de chances. Fonte: elaboração dos autores.

### Papel dos fatores socioeconômicos

Além de corroborar a literatura existente, nosso estudo avança a discussão das disparidades e o papel do acesso como um fator de risco para eventos cardiovasculares.^22^ O índice socioeconômico (IFDM) em nosso estudo não mostrou uma correlação estatisticamente significativa com os desfechos cardiovasculares na população global com AR (p > 0,05). Isso contrasta com outros estudos que indicam que 80% do risco cardiovascular na AR surge em países com nível socioeconômico baixo a médio,^23^ mas menos de 20% dos estudos clínicos incluem nível socioeconômico em seus modelos.^20^

### Pontos fortes e limitações

O presente estudo apresenta vários pontos fortes, incluindo um grande tamanho de amostra nacional e uma análise abrangente de fatores clínicos e socioeconômicos, fornecendo insights valiosos sobre os riscos cardiovasculares enfrentados por pacientes com AR no Brasil. O uso do DATASUS permitiu a inclusão de uma população diversificada de pacientes em vários contextos de assistência médica. No entanto, o estudo se concentra em pacientes que recebem medicamentos de alta complexidade para AR grave, o que pode não representar aqueles com doença inicial ou de leve a moderada. Algumas limitações incluem variações potenciais na precisão dos dados, fatores de confusão residuais não levados em consideração (por exemplo, dieta, atividade física, predisposições genéticas, adesão à medicação) e ausência de dados clínicos detalhados (por exemplo, escores de atividade da doença e resultados laboratoriais). Mudanças nas práticas clínicas e na qualidade da assistência médica ao longo do período do estudo também podem ter influenciado os resultados. Apesar dessas limitações, os pontos fortes deste estudo ajudam a mitigar essas preocupações. Pesquisas futuras podem se concentrar em pacientes com AR em estágio inicial ou aqueles com gravidade da doença leve a moderada para expandir ainda mais esses achados.

### Implicações para pesquisas e políticas futuras

A relevância do presente estudo está na exploração de aspectos cardiovasculares em uma grande amostra nacional de pacientes com AR no Brasil. Nossos achados reforçam a premissa de que um melhor controle da inflamação se traduz em uma redução dos desfechos cardiovasculares em pacientes com AR. Como uma perspectiva futura, essa evidência pode orientar a pesquisa de eficácia comparativa com as opções terapêuticas analisadas aqui, usando eventos cardiovasculares como medidas de desfecho. Essa abordagem também pode incluir estudos comparativos de custos, que podem ser úteis para a elaboração de políticas públicas no Brasil e no contexto da carga global da doença.

Além disso, os achados do nosso estudo podem informar os provedores de saúde e formuladores de políticas sobre a importância de tratamentos personalizados para pacientes com AR, considerando fatores clínicos e socioeconômicos para otimizar os desfechos de saúde cardiovascular.

## Conclusão

Nosso estudo demonstra que fatores de risco cardiovascular tradicionais, fatores socioeconômicos e fatores relacionados à inflamação impactam significativamente os desfechos cardiovasculares em pacientes com AR grave recebendo tratamentos biológicos de alta complexidade. Esses achados destacam a importância do manejo abrangente do risco cardiovascular e os benefícios do controle da inflamação por meio de DMARDs apropriados, particularmente DMARDs biológicos. Embora a grande amostra nacional do estudo e a análise detalhada forneçam conhecimentos valiosos, pesquisas futuras devem se concentrar em pacientes com AR em estágio inicial ou com doença leve a moderada para entender melhor os riscos cardiovasculares em diferentes estágios da doença e considerar fatores de confusão residuais e dados clínicos detalhados para melhorar a compreensão dessas relações.
