# Supplementary material for: A Frequência de Doenças Cardiovasculares na Artrite Reumatoide no Brasil: Estudo de Coorte de 10 Anos com Bancos de Dados do DATASUS
Source: Arq Bras Cardiol. 2025 Feb 27;122(2):e20240313. [Article in Portuguese] doi: 10.36660/abc.20240313 (PMC12080704; doi:10.36660/abc.20240313)
Supplement: Supplementary file 1 [file 0066-782X-abc-122-2-e20240313-suppl01.pdf]

WEB SUPPLEMENTAL MATERIAL

Title: The Prevalence of Cardiovascular Disease in Rheumatoid Arthritis in Brazil: Lessons Learned From 10-Years of DATASUS Cohort

Index of Contents

Section 1. Demographic and Clinical Information in administrative databases 2
Section 2. Cardiovascular outcomes 7
Section 3. Algorithm equation for Cardiovascular outcomes 8
Section 4. Socio-Economic Information - FIRJAN Municipal Development Index 9

Section 1. Demographic and Clinical Information in administrative databases

DATASUS is the health information system that was created in 2008; its databases are composed to reflect patient care. Each patient has a personal identification number, which enables the integration of the database used for this study. In addition to the identification number, for each care provided such as medication, professional visit, day of hospitalization and so on, a procedure code is designed with specific numbering to enter the numbered field of the medical bills audit form. Some SUS information is registered in the code of procedures, and other information, referring to procedures, only enters after auditing medical bills, carried out by physicians. DATASUS databases include those generated by outpatient care forms, hospital care forms, epidemiological surveillance data, death certificate forms, and so on. The forms aim to capture the information to be entered into the system. They include registration data, such as name, date of birth, gender, residence, such as clinical data, as well as the technologies incorporated for the care of the respective pathologies. The technologies used in patient care are identified from procedure codes. These codes are used to pay the bills related to resources consumed in each municipality or region. Once the accounts are audited and approved, they become part of the DATASUS databases.

a) APAC (High-Cost Procedure Authorization): Included are date of birth, gender, state, city, ICD, rheumatic disease code, treatment phase, (initial or return), immunosuppressive treatment, dialysis (yes or no) and professional visits during the study period;

b) AIH (Hospital Admission Authorization): The date of hospitalization, discharge and birth, gender, city, length of hospitalization, ICD reason for hospitalization, date and reason of death, when applicable, are included. There is also the type of hospitalization (urgency or emergency) and reason for dehospitalization (cure or death);

c) BPAI (Individualized Ambulatory Production Bulletin): The BPAI form includes patient data such as CNS, gender, date of birth. Race, color and ethnicity, in addition to the code of the municipality of residence. The BPA-I also includes the CNES of the performing establishment, procedure code, quantity of procedures performed, date and respective ICD. The scope of the extraction is national, carried out between 2008 and 2018.

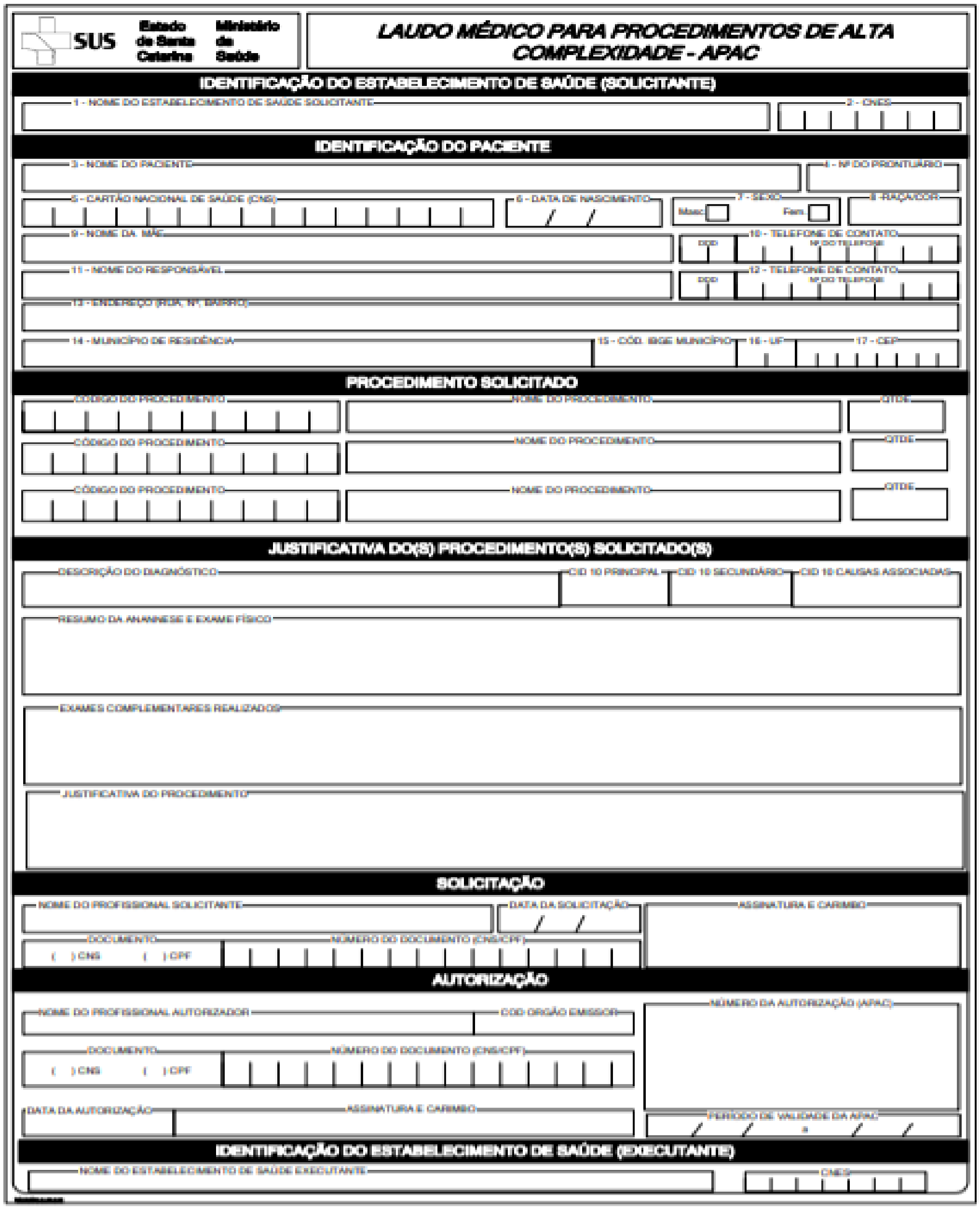

SUS | Estado de Santa Catarina | Ministério da Saúde

LAUDO MÉDICO PARA PROCEDIMENTOS DE ALTA COMPLEXIDADE - APAC

IDENTIFICAÇÃO DO ESTABELECIMENTO DE SAÚDE (SOLICITANTE)

1 - NOME DO ESTABELECIMENTO DE SAÚDE SOLICITANTE | 2 - CNES

IDENTIFICAÇÃO DO PACIENTE

3 - NOME DO PACIENTE | 4 - Nº DO PRONTUÁRIO

5 - CARTÃO NACIONAL DE SAÚDE (CNS) | 6 - DATA DE NASCIMENTO | 7 - SEXO Masc. Fem. | 8 - RAÇA/COR

9 - NOME DA MÃE | 10 - TELEFONE DE CONTATO DDD Nº DO TELEFONE

11 - NOME DO RESPONSÁVEL | 12 - TELEFONE DE CONTATO DDD Nº DO TELEFONE

13 - ENDEREÇO (RUA, Nº, BAIRRO)

14 - MUNICÍPIO DE RESIDÊNCIA | 15 - CÓD. IBGE MUNICÍPIO | 16 - UF | 17 - CEP

PROCEDIMENTO SOLICITADO

CÓDIGO DO PROCEDIMENTO | NOME DO PROCEDIMENTO | QTDE

CÓDIGO DO PROCEDIMENTO | NOME DO PROCEDIMENTO | QTDE

CÓDIGO DO PROCEDIMENTO | NOME DO PROCEDIMENTO | QTDE

JUSTIFICATIVA DO(S) PROCEDIMENTO(S) SOLICITADO(S)

DESCRIÇÃO DO DIAGNÓSTICO | CID 10 PRINCIPAL | CID 10 SECUNDÁRIO | CID 10 CAUSAS ASSOCIADAS

RESUMO DA ANANNESE E EXAME FÍSICO

EXAMES COMPLEMENTARES REALIZADOS

JUSTIFICATIVA DO PROCEDIMENTO

SOLICITAÇÃO

NOME DO PROFISSIONAL SOLICITANTE | DATA DA SOLICITAÇÃO | ASSINATURA E CARIMBO

DOCUMENTO ( ) CNS ( ) CPF | NÚMERO DO DOCUMENTO (CNS/CPF)

AUTORIZAÇÃO

NOME DO PROFISSIONAL AUTORIZADOR | COD ORGÃO EMISSOR | NÚMERO DA AUTORIZAÇÃO (APAC)

DOCUMENTO ( ) CNS ( ) CPF | NÚMERO DO DOCUMENTO (CNS/CPF)

DATA DA AUTORIZAÇÃO | ASSINATURA E CARIMBO | PERÍODO DE VALIDADE DA APAC / / a / /

IDENTIFICAÇÃO DO ESTABELECIMENTO DE SAÚDE (EXECUTANTE)

NOME DO ESTABELECIMENTO DE SAÚDE EXECUTANTE | CNES

Figure S1-A. Demonstration of APAC, AIH and BPA-I forms.

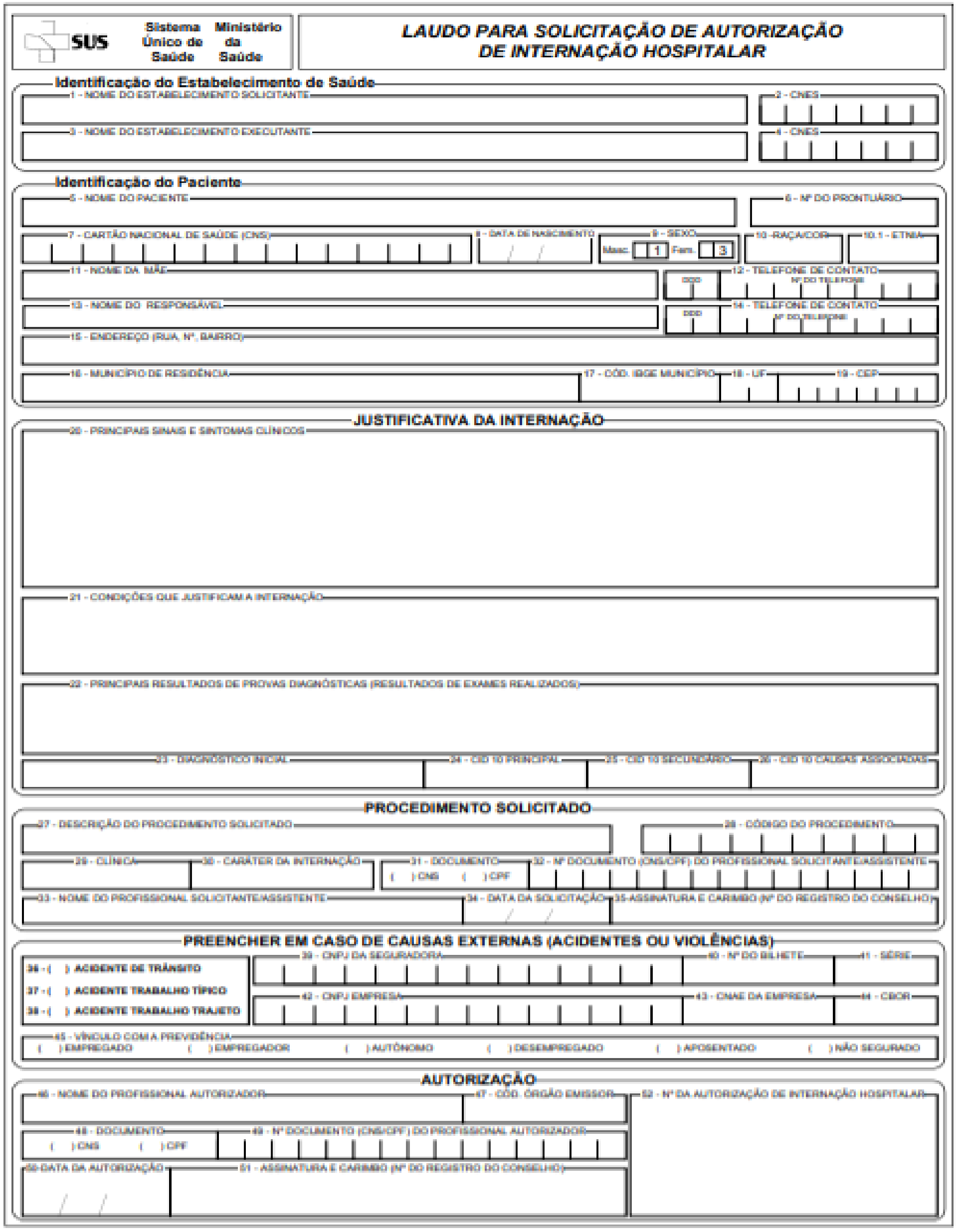

SUS Sistema Único de Saúde | Ministério da Saúde

LAUDO PARA SOLICITAÇÃO DE AUTORIZAÇÃO DE INTERNAÇÃO HOSPITALAR

Identificação do Estabelecimento de Saúde

1 - NOME DO ESTABELECIMENTO SOLICITANTE
2 - CNES
3 - NOME DO ESTABELECIMENTO EXECUTANTE
4 - CNES

Identificação do Paciente

5 - NOME DO PACIENTE
6 - Nº DO PRONTUÁRIO
7 - CARTÃO NACIONAL DE SAÚDE (CNS)
8 - DATA DE NASCIMENTO
9 - SEXO Masc. 1 Fem. 3
10 - RAÇA/COR
10.1 - ETNIA
11 - NOME DA MÃE
12 - TELEFONE DE CONTATO DDD Nº DO TELEFONE
13 - NOME DO RESPONSÁVEL
14 - TELEFONE DE CONTATO DDD Nº DO TELEFONE
15 - ENDEREÇO (RUA, Nº, BAIRRO)
16 - MUNICÍPIO DE RESIDÊNCIA
17 - CÓD. IBGE MUNICÍPIO
18 - UF
19 - CEP

JUSTIFICATIVA DA INTERNAÇÃO

20 - PRINCIPAIS SINAIS E SINTOMAS CLÍNICOS
21 - CONDIÇÕES QUE JUSTIFICAM A INTERNAÇÃO
22 - PRINCIPAIS RESULTADOS DE PROVAS DIAGNÓSTICAS (RESULTADOS DE EXAMES REALIZADOS)
23 - DIAGNÓSTICO INICIAL
24 - CID 10 PRINCIPAL
25 - CID 10 SECUNDÁRIO
26 - CID 10 CAUSAS ASSOCIADAS

PROCEDIMENTO SOLICITADO

27 - DESCRIÇÃO DO PROCEDIMENTO SOLICITADO
28 - CÓDIGO DO PROCEDIMENTO
29 - CLÍNICA
30 - CARÁTER DA INTERNAÇÃO
31 - DOCUMENTO ( ) CNS ( ) CPF
32 - Nº DOCUMENTO (CNS/CPF) DO PROFISSIONAL SOLICITANTE/ASSISTENTE
33 - NOME DO PROFISSIONAL SOLICITANTE/ASSISTENTE
34 - DATA DA SOLICITAÇÃO
35 - ASSINATURA E CARIMBO (Nº DO REGISTRO DO CONSELHO)

PREENCHER EM CASO DE CAUSAS EXTERNAS (ACIDENTES OU VIOLÊNCIAS)

36 - ( ) ACIDENTE DE TRÂNSITO
37 - ( ) ACIDENTE TRABALHO TÍPICO
38 - ( ) ACIDENTE TRABALHO TRAJETO
39 - CNPJ DA SEGURADORA
40 - Nº DO BILHETE
41 - SÉRIE
42 - CNPJ EMPRESA
43 - CNAE DA EMPRESA
44 - CBOR
45 - VÍNCULO COM A PREVIDÊNCIA ( ) EMPREGADO ( ) EMPREGADOR ( ) AUTÔNOMO ( ) DESEMPREGADO ( ) APOSENTADO ( ) NÃO SEGURADO

AUTORIZAÇÃO

46 - NOME DO PROFISSIONAL AUTORIZADOR
47 - CÓD. ÓRGÃO EMISSOR
52 - Nº DA AUTORIZAÇÃO DE INTERNAÇÃO HOSPITALAR
48 - DOCUMENTO ( ) CNS ( ) CPF
49 - Nº DOCUMENTO (CNS/CPF) DO PROFISSIONAL AUTORIZADOR
50 - DATA DA AUTORIZAÇÃO
51 - ASSINATURA E CARIMBO (Nº DO REGISTRO DO CONSELHO)

Figure S1-B. Demonstration of AIH form.

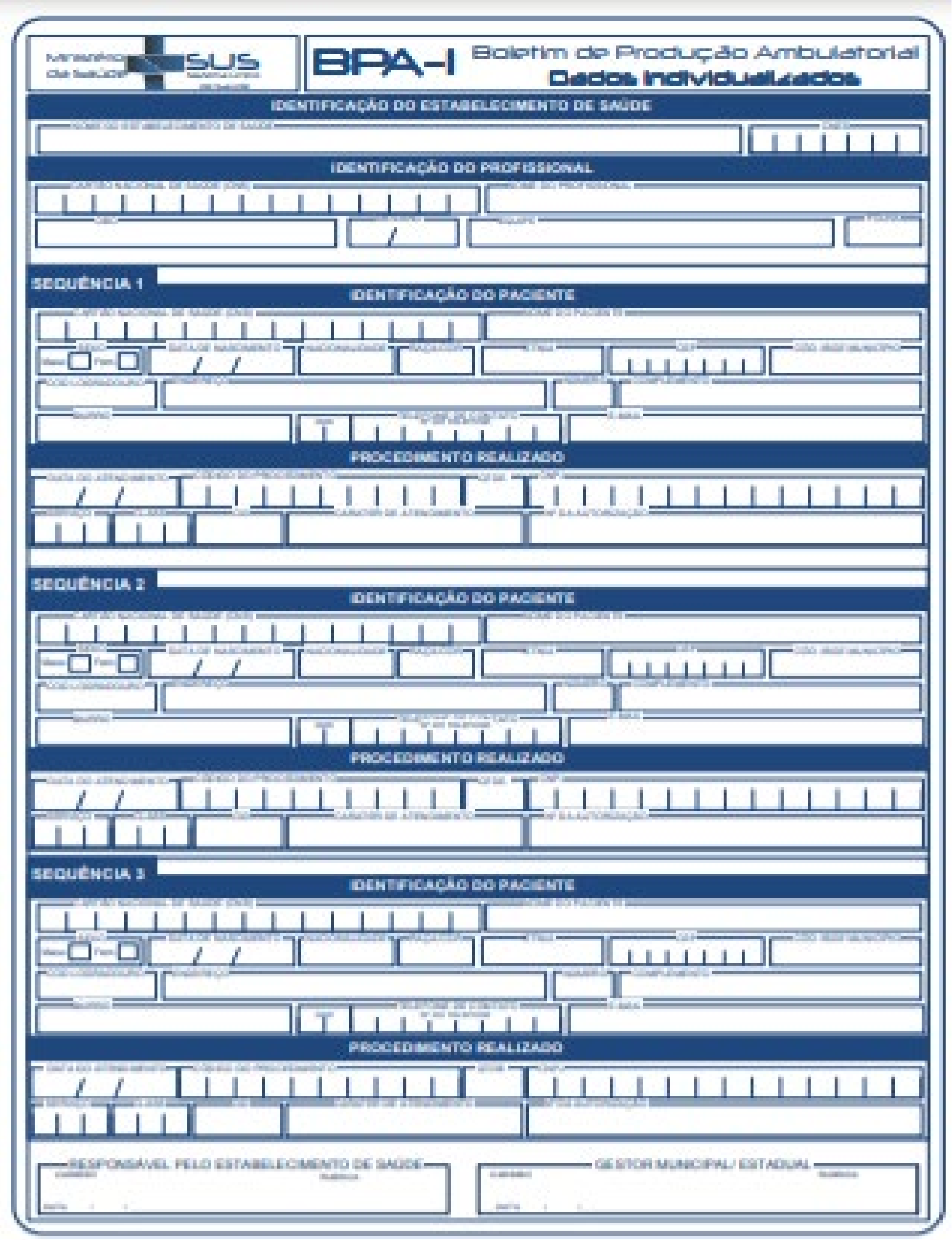

BPA-I Boletim de Produção Ambulatorial
Dados Individualizados

IDENTIFICAÇÃO DO ESTABELECIMENTO DE SAÚDE

IDENTIFICAÇÃO DO PROFISSIONAL

SEQUÊNCIA 1

IDENTIFICAÇÃO DO PACIENTE

PROCEDIMENTO REALIZADO

SEQUÊNCIA 2

IDENTIFICAÇÃO DO PACIENTE

PROCEDIMENTO REALIZADO

SEQUÊNCIA 3

IDENTIFICAÇÃO DO PACIENTE

PROCEDIMENTO REALIZADO

RESPONSÁVEL PELO ESTABELECIMENTO DE SAÚDE

GESTOR MUNICIPAL/ ESTADUAL

Figure S1-C. Demonstration of BPA-I form.

Section 2. Cardiovascular outcomes

This is composed from data found in the APAC, relating them to the name and/or ICD of the procedure performed that corresponds to the outcome under evaluation. Furthermore, covered high-cost medication procedures are used to treat the outcome in question. Underneath, we will explain the construction process of this equation. The present study does not consider cardiovascular outcomes that occurred prior to the inclusion of the patient in the baseline of the study. Outcomes are searched in administrative databases during the 48-month patient segment included in the study from the index date.

For myocardial ischemic disease, acute myocardial infarction and acute myocardial ischemic syndromes are included, for heart failure, insufficiency with reduced or preserved ejection fraction is not separated. For cerebrovascular accident (CVA), transient, definitive, or ischemic stroke were included.

Section 3. Algorithm equation for Cardiovascular outcomes

Acute CAD – Unstable Coronary Syndrome - Myocardial Infarction - Recurrent Myocardial Infarction: [presence of ICD (I-20—I21 − I22 and I24) + (presence of diagnostic procedure associated with ICD (I-20-I21 − I22 and I24) covered by SUS) or (presence of therapeutic procedure associated with the ICD (I-20-I21-I22 and I24) covered by SUS)].

Heart Failure (HF): [(presence of ICD I-50) + (presence of diagnostic procedure associated with ICD I-50 covered by SUS) or (presence of therapeutic procedure associated with ICD I-50 covered by SUS)].

Stroke (CVA): [(presence of ICD I-64-G-45) + (presence of diagnostic procedure associated with ICD I-64-G-45) or (presence of therapeutic procedure associated with ICD I-64-G-45)].

Therefore, the cardiovascular outcomes selected for evaluation will be acute ischemic coronary syndromes, global and cardiovascular mortality, heart failure and stroke, including data related to the ICD of the cardiovascular outcome and diagnostic and/or therapeutic procedures related to them, for their characterization.

Index date: This will be the date of the first registered DMARD entry information, for the patient included in the study, which is in the database.

Section 4. Socio-Economic Information - FIRJAN Municipal Development Index

This survey was based on information from the Firjan Municipal Development Index (IFDM), which is a synthetic indicator of municipal quality of life. Employment/ Income, Education and Health. This indicator is build based entirely on official public statistics, made available by the ministries of Labor, Education and Health in Brazil. It can be read, in general, or it can also be analyzed by sector, in terms of employment/income, health and education. The index ranges from 0 (minimum) to 1 point (maximum) to classify the level of each location into four categories: low (from 0 to 0.4), regular (0.4 to 0.6) moderate (from 0.6 to 0.8) and high (0.8 to 1) development. That is, the closer to 1, the greater the development of the location. This index gives us the idea of the socioeconomic development index of the municipality or region of the country analyzed, allowing us to associate aspects related to development in the global sphere, income, employment, health and education with the quality of life and aspects related to the local health under evaluation.

Table S1. Sample distribution regarding social, demographic, and traditional risk factors for CV events in relation to RA treatment at baseline.

Treatment	Add-on	BC	SC	BS	SBS	SS	SSB
N	855	334	863	140	110	1483	446
Female	687 (80.4%)	217 (65.0%)	730 (84.6%)	98 (70.0%)	87 (79.1%)	1232 (83.1%)	347 (77.8%)
Male	168 (19.6%)	117 (35.0%)	133 (15.4%)	42 (30.0%)	23 (20.9%)	251 (16.9%)	99 (22.2%)
Average Age	52.12 (12.31)	53.18 (13.27)	54.49 (12.54)	48.55 (12.86)	52.24 (11.72)	56.64 (12.90)	52.69 (12.44)
< 40 years old	154 (18.0%)	68 (20.4%)	130 (15.1%)	38 (27.1%)	16 (14.5%)	173 (11.7%)	69 (15.5%)
40 to 60 years old	492 (57.5%)	161 (48.2%)	458 (53.1%)	77 (55.0%)	69 (62.7%)	700 (47.2%)	253 (56.7%)
60 to 70 years old	169 (19.8%)	73 (21.9%)	201 (23.3%)	20 (14.3%)	17 (15.5%)	422 (28.5%)	91 (20.4%)
70 to 80 years old	35 (4.1%)	27 (8.1%)	62 (7.2%)	5 (3.6%)	8 (7.3%)	155 (10.5%)	32 (7.2%)
> 80 years old	5 (0.6%)	5 (1.5%)	12 (1.4%)	0 (0.0%)	0 (0.0%)	33 (2.2%)	1 (0.2%)
Caucasian	439 (51.3%)	175 (52.4%)	381 (44.1%)	75 (53.6%)	64 (58.2%)	794 (53.5%)	210 (47.1%)
Afro Descendent	160 (18.7%)	55 (16.5%)	188 (21.8%)	30 (21.4%)	17 (15.4%)	297 (20.1%)	87 (19.5%)
South East Region	460 (53.8%)	195 (58.4%)	534 (61.9%)	78 (55.7%)	81 (73.6%)	855 (57.7%)	218 (48.9%)
North East Region	102 (11.9%)	44 (13.2%)	75 (8.7%)	25 (17.9%)	5 (4.5%)	159 (10.7%)	64 (14.3%)
North Region	14 (1.6%)	7 (2.1%)	21 (2.4%)	3 (2.1%)	5 (4.5%)	13 (0.9%)	28 (6.3%)
South Region	232 (27.1%)	59 (17.7%)	190 (22.0%)	28 (20.0%)	15 (13.6%)	412 (27.8%)	114 (25.6%)
Mid-West Region	47 (5.5%)	29 (8.7%)	43 (5.0%)	6 (4.3%)	4 (3.6%)	44 (3.0%)	22 (4.9%)
IFDM * >0,8	378 (44.2%)	139 (41.6%)	414 (48.0%)	52 (37.1%)	64 (58.2%)	774 (52.2%)	189 (42.4%)
IFDM employment - income 0,6-0,7	348 (40.7%)	149 (44.6%)	329 (38.1%)	64 (45.7%)	64 (58.2%)	605 (40.8%)	197 (44.2%)
IFDM health >0,8	676 (79.1%)	284 (85.0%)	704 (81.6%)	125 (89.3%)	95 (86.4%)	1211 (81.7%)	348 (78.0%)
IFDM education >0,8	590 (69.0%)	241 (72.2%)	637 (73.8%)	94 (67.1%)	88 (80.0%)	1092 (73.6%)	289 (64.8%)
Risk Factor: 0	834 (97.5%)	329 (98.5%)	855 (99.1%)	136 (97.1%)	106 (96.4%)	1409 (95.0%)	433 (97.1%)
Risk Factor: 1+	21 (2.5%)	5 (1.5%)	8 (0.9%)	4 (2.9%)	4 (3.6%)	74 (5.0%)	13 (2.9%)

Add-on addition of Biological to Synthetic DMARD
BC - Biological Constant
CS - Constant Synthetic
[4] SB - Swift Biological
[5] SBS - Swift Biological to Synthetic
[6] SS - Swift Synthetic
[7] SSB - Swift Synthetic to Biological
[8] IFDM - Firjan Index of Municipal Development

Table S2. Detailed definition of treatment categorization of the rheumatoid arthritis patient population.

**Add-on**	When at least one medication is added during study follow-up
**Biological Constant (BC)**	When the same biological drug remained constant throughout the study follow-up
**Synthetic Constant (SC)**	When the same synthetic drug remained constant throughout the study follow-up
**Biological Swift (BS)**	When there is a change from a given biological medicine to another category of biological medicine during the follow-up of the study
**Biological -> Synthetic Swift (BSS)**	When there is a change from a given biological medicine to another category of synthetic medicine during the follow-up of the study
**Synthetic Swift (SS)**	When there is a change from a given synthetic drug to another category of synthetic drug during the study follow-up
**Synthetic -> Biological Swift (SBS)**	When there is a change from a given synthetic drug to another category of biological drug during the study follow-up

Table S3. Classification levels of the FIRJAN Municipal Development Index.

**Low**	0 to 0.4
**Regular**	0.6 to 0.8
**Moderate**	0.6 to 0.8
**High**	0.8 to 1

Table S4. Distribution of the population according to the distribution of specific drugs for RA throughout the study.

RA Medication	Population (4,231)	(%)
**Add-on**	855	20.2%
**Biological Constant**	334	7.9%
**Synthetic Constant**	863	20.4%
**Biological Swift**	140	3.3%
**Biological + Synthetic Swift**	110	2.6%
**Synthetic Swift**	1,483	35.1%
**Synthetic + Biological Swift**	446	10.5%
**Biological Group**	1,885	44.6%
**Synthetic Group**	3,757	88.8%

Biological Group is the sum of the groups with biological medication.
Synthetic Group is the sum of the groups with synthetic medication.

Table S5. Social, demographic, and clinical distribution of the population with cardiovascular outcomes over 48 months.

Characteristics		Number of patients
Gender	Female	142 (77.2%)
	Male	42 (22.8%)
Age	Average Age	59.93 (12.23)
Race	Caucasian	123 (66.8%)
	Afro descendent	42 (42.8%)
	Asian	8 (4.3%)
Age group	< 40	15 (8.2%)
	40 |--| 60	76 (41.3%)
	60 |--| 70	59 (32.1%)
	70 |--| 80	26 (14.1%)
	80 >	8 (4.3%)
Region	Midwest	5 (2.7%)
	North East	18 (9.8%)
	North	3 (1.6%)
	Southeast	104 (56.5%)
	South	54 (29.3%)
General IFDM	0,8 >	97 (52.7%)
IFDM employment/income	0,6 a 0,7	75 (40.8%)
IFDM health	0,8 >	153 (83.2%)
IFDM education	0,8 >	132 (71.7%)
Risk Factors	0	170 (92.4%)
	1	14 (7.6%)

N= 184 (patients with cardiovascular outcomes)
Number of cardiovascular outcomes = 198
IFDM - FIRJAN Municipal Development Index (predominant)

Table S6. IFDM according to regional distribution of Brazil.

	n	Loss	Average	Standard Deviation
**Midwest**				
IFDM	195	36	0.77	0.052
Employment/ Income	195	36	0.63	0.090
Education	195	36	0.82	0.044
Health	195	36	0.87	0.058
**North East**				
IFDM	474	0	0.71	0.070
Employment/ Income	474	0	0.61	0.132
Education	474	0	0.72	0.067
Health	474	0	0.79	0.083
**North**				
IFDM	91	0	0.68	0.076
Employment/ Income	91	0	0.60	0.113
Education	91	0	0.71	0.071
Health	91	0	0.72	0.090
**Southeast**				
IFDM	2421	4	0.80	0.058
Employment/ Income	2421	4	0.61	0.092
Education	2421	0	0.90	0.063
Health	2421	0	0.87	0.065
**South**				
IFDM	1050	2	0.79	0.059
Employment/ Income	1050	0	0.65	0.103
Education	1050	2	0.82	0.067
Health	1050	0	0.89	0.058

Table S7-A. Comparison of proportions between different treatment models and Odd-Ratio for cardiovascular outcomes.

	Add-on	BC	SC	BS	BSS	SS
**BC**	1	-	-	-	-	-
**SC**	1	1	-	-	-	-
**BS**	1	1	1	NA	-	-
**BSS**	1	1	1	1	-	-
**SS**	0,1619	0,8791	0,0024	0,9597	1	-
**SBS**	1	1	1	1	1	1

Table S7-B. Comparison of proportions between different treatment models and Odd-Ratio for cardiovascular outcomes.

	SC	Add-On	SS	SBS	BC	BSS	BS
**SC**	1	0.786 [0.444; 1.4]	0.433 [0.273; 0.668]	1.13 [0.553; 2.46]	0.856 [0.395; 2.02]	1.3 [0.351; 9.14]	1.26 [0.411; 5.74]
**Add-On**	1.27 [0.716; 2.25]	1	0.551 [0.338; 0.871]	1.44 [0.691; 3.18]	1.09 [0.494; 2.61]	1.65 [0.442; 11.7]	1.61 [0.517; 7.35]
**SS**	**2.31 [1.5; 3.66]**	1.81 [1.15; 2.96]	1	2.61 [1.4; 5.35]	1.98 [0.99; 4.42]	3 [0.855; 20.6]	2.92 [1.01; 12.8]
**SBS**	0.886 [0.406; 1.81]	0.696 [0.315; 1.45]	0.383 [0.187; 0.716]	1	0.758 [0.293; 2.01]	1.15 [0.275; 8.59]	1.12 [0.318; 5.46]
**BC**	1.17 [0.494; 2.53]	0.919 [0.384; 2.02]	0.506 [0.226; 1.01]	1.32 [0.497; 3.41]	1	1.52 [0.345; 11.6]	1.47 [0.396; 7.41]
**BSS**	0.77 [0.109; 2.85]	0.605 [0.0855; 2.26]	0.333 [0.0486; 1.17]	0.869 [0.116; 3.63]	0.66 [0.0862; 2.9]	1	0.973 [0.106; 6.93]
**BS**	0.791 [0.174; 2.43]	0.622 [0.136; 1.94]	0.342 [0.0779; 0.992]	0.893 [0.183; 3.15]	0.678 [0.135; 2.52]	1.03 [0.144; 9.47]	1

Table S8. Pairing 1:15 of patients with and without cardiovascular outcomes considering adjusted variables.

	Characteristics	No	Yes	p
**Patients**	No.	920	184	-
**Gender**	Female	746 (81.1%)	142 (77.2%)	0.263
	Male	174 (18.9%)	42 (22.8%)	
**Age Group**	<- 40	75 (8.2%)	15 (8.2%)	0.999
	40 |--| 60	380 (41.3%)	76 (41.3%)	
	60 |--| 70	295 (32.1%)	59 (32.1%)	
	70 |--| 80	134 (14.6%)	26 (14.1%)	
	80 >-	36 (3.9%)	8 (4.3%)	
**General IFDM**	Without IFDM	15 (1.6%)	1 (0.5%)	0.543
	0.6 <	14 (1.5%)	2 (1.1%)	
	0.6 -- 0.7	108 (11.7%)	16 (8.7%)	
	0.7 -- 0.8	322 (35.0%)	68 (37.0%)	
	0.8 >	461 (50.1%)	97 (52.7%)	
**IFDM Employment /Income**	Without IFDM	14 (1.5%)	1 (0.5%)	0.818
	0.6 <	225 (24.5%)	47 (25.5%)	
	0.6 -- 0.7	389 (42.3%)	75 (40.8%)	
	0.7 -- 0.8	291 (31.6%)	61 (33.2%)	
	0.8 >	1 (0.1%)	0 (0.0%)	
**IFDM Health**	Without IFDM	12 (1.3%)	0 (0.0%)	0.547
	0.6 <	12 (1.3%)	2 (1.1%)	
	0.6 -- 0.7	32 (3.5%)	7 (3.8%)	
	0.7 -- 0.8	127 (13.8%)	22 (12.0%)	
	0.8 >	737 (80.1%)	153 (83.2%)	
**IFDM Education**	Without IFDM	13 (1.4%)	0 (0.0%)	0.388
	0.6 <	8 (0.9%)	3 (1.6%)	
	0.6 -- 0.7	63 (6.8%)	10 (5.4%)	
	0.7 -- 0.8	181 (19.7%)	39 (21.2%)	
	0.8 >	655 (71.2%)	132 (71.7%)	
**Risk Factor**	0	860 (93.5%)	170 (92.4%)	0.706
	1+	60 (6.5%)	14 (7.6%)	
**Treatment Type**	Add on	155 (16.8%)	26 (14.1%)	<0.001
	Biological Constant	59 (6.4%)	9 (4.9%)	
	Synthetic Constant	220 (23.9%)	29 (15.8%)	
	Biological Swift	30 (3.3%)	3 (1.6%)	
	Biological Synthetic Swift	21 (2.3%)	2 (1.1%)	
	Synthetic Swift	340 (37.0%)	104 (56.5%)	

	Synthetic Biological Swift	95 (10.3%)	11 (6.0%)	
**Region**	Midwest	46 (5.0%)	5 (2.7%)	0.295
	Northeast	114 (12.4%)	18 (9.8%)	
	North	26 (2.8%)	3 (1.6%)	
	Southeast	509 (55.3%)	104 (56.5%)	
	South	225 (24.5%)	54 (29.3%)	

Table S9. Definition of covariates prepared for the study.

**Diabetes**	with ICD E10 and E11 with PCDT for diabetes (Clinical Protocols and Therapeutic Guidelines)
**Hypertension**	with ICD I10 to I15, and PCDT for Hypertension
**Dyslipidemia**	with ICD E 78 and PCDT for Dyslipidemia
**Smoking**	with ICD F 17 to Z 72 and PCDT suitable for smoking
**Obesity**	with ICD E 65 to E 68 and PCDT suitable for obesity

Table S10. Distribution of traditional cardiovascular risk factors in all patients included and in the patients with cardiovascular outcomes.

**All patients included with RA**<br>n= 4231		**Patients with cardiovascular outcomes**<br>n= 184
Cardiovascular Risk factors<br>n= 129 (3%)		Cardiovascular Risk factors<br>n= 14 (7.6%)
Hipertension – 38 (0.9%)	ICD+PCDT	Hipertension – 7 (3.8%)
Diabetes – 19 (0.4%)	ICD+PCDT	Diabetes – 3 (1.6%)
Dislipidemia -58 (1.4%)	ICD+PCDT	Dislipidemia – 2 (1.1%)
Smoking – 16 (0.4%)	ICD+PCDT	Smoking – 2 (1.1%)
Obesity – 2	ICD+PCDT	Obesity – 0
