# Supplementary material for: A Frequência de Doenças Cardiovasculares na Artrite Reumatoide no Brasil: Estudo de Coorte de 10 Anos com Bancos de Dados do DATASUS
Source: Arq Bras Cardiol. 2025 Feb 27;122(2):e20240313. [Article in Portuguese] doi: 10.36660/abc.20240313 (PMC12080704; doi:10.36660/abc.20240313)
Supplement: Translation

# Translation: The Frequency of Cardiovascular Diseases in Rheumatoid Arthritis in Brazil: 10-year Cohort Study with DATASUS Databases

**DOI:** 10.36660/abc.20240313i

**Authors:** Roberto Gamarski, Fernando Ferreira de Castro, Jose Antonio Sena do Nascimento, Mirhelen Mendes de Abreu

**Affiliations:** 1 Brasil Universidade Federal do Rio de Janeiro, Rio de Janeiro, RJ – Brasil

**Keywords:** Rheumatoid Arthritis, Cardiovascular Diseases, Health Care Outcome Assessment, Antirheumatic Agents

## Abstract

**Background:**

Cardiovascular outcomes in patients with rheumatoid arthritis (RA) have been extensively explored in the literature concerning biological factors.

**Objectives:**

This nationwide retrospective cohort study aimed to explore the distribution of cardiovascular events in patients with RA in Brazil, assisted by the Unified Health System (SUS), and to identify factors associated with these outcomes.

**Methods:**

Patients aged ≥ 18 years were identified from the Brazilian Unified Health System Database (DATASUS) through RA ICD-10 codes and their therapeutic procedures according to SUS guidelines. RA patients treated with disease-modifying antirheumatic drugs (DMARDs) were categorized as biological users and non-biological (synthetic) users. Cardiovascular outcomes, including acute coronary artery disease (ACAD), heart failure, and cerebrovascular accident (CVA), were analyzed. Patients were also categorized based on treatment patterns (switch or constant users). Socioeconomic status was assessed using the FIRJAN Municipal Development Index (IFDM). Descriptive analyses identified population distribution and cardiovascular outcomes, and multiple logistic regression explored associated factors. The statistical significance adopted was p < 0.05.

**Results:**

Among the 4,321 patients with RA treated with DMARDS, 198 cardiovascular outcomes (4.68%) were identified. The majority were female (3,398 [80.3%]) with a mean age of 54.2 (standard deviation 12.8) years, predominantly from the Southeast Region (2,421 [57.2%]). The predominant overall IFDM was > 0.8 (47.5%). Advanced age, the presence of cardiovascular risk factors, and the use of synthetic DMARDs were associated with cardiovascular outcomes.

**Conclusion:**

Cardiovascular outcomes in patients with RA are common and are associated with age, comorbidities, and the drugs used for treatment.

## Introduction

Cardiovascular events are a significant concern in the etiopathogenesis and natural history of rheumatoid arthritis (RA).^1^Elevated inflammatory markers in patients with RA are associated with increased cardiovascular mortality and greater disease activity.^2 , 3^While traditional cardiovascular risk factors such as hypertension and diabetes are well known, the role of RA treatments, particularly biological disease-modifying anti-rheumatic drugs (DMARDs), in reducing cardiovascular risks has also been highlighted in the literature.^4^

This study utilizes data from DATASUS, the health information system of the Brazilian Unified Health System (SUS) of the Brazilian Ministry of Health,^5^which covers 80% of the Brazilian population. By exploring data from this comprehensive system, our study aims to describe the distribution of cardiovascular disease in patients with RA treated with DMARDs in Brazil and identify the factors associated with these outcomes. The DMARDs evaluated are described in the methodology.

Given that socio-environmental indicators like climatic conditions, pollution, and human development index have been less explored,^6 , 7^the study evaluated the FIRJAN Municipal Development Index (IFDM), a synthetic indicator of municipal quality of life, which is detailed in the methodology and Web Supplemental Material . Specifically, we aim to understand the interaction between synthetic and biological DMARDs in preventing cardiovascular outcomes ( Web Supplemental Material, Section 1 ), describe the distribution of cardiovascular disease in this group of patients, and identify the role of contributors to the outcomes, such as comorbidities, demographic, and socioeconomic factors. This study seeks to fill gaps in current knowledge and provide insights into how these treatments impact cardiovascular health in patients with RA in the Brazilian context.

The key problem to be addressed is to understand the frequency of cardiovascular outcomes in patients with RA. Some factors affect these outcomes and are referred to as multifactorial in the literature; therefore, we seek to identify the relevance of these factors, such as demographic, social, and clinical variables. The results of this project may contribute to the development of scientific knowledge on the interaction between cardiovascular disease and RA by identifying the role of these multifactorial variables.

## Methods

The methodological procedures began with the integration of administrative databases from DATASUS, which took place in three phases: (1) construction and validation of the algorithms used to identify the subjects in the previously extracted database, (2) construction and validation of the algorithms used to identify demographic and clinical variables, (3) application of the algorithms and testing of the model to analyze the association between these variables and cardiovascular outcomes.

### Outline

This study was an observational longitudinal retrospective cohort with nationwide data extraction from DATASUS between 2008 and 2018 ( Web Supplemental Material, Section 2 ). Patients were included in the study from 2009 to 2017, and the study period for each patient was 4 years (48 months), with follow-up censored due to death, loss of follow-up, or completion of the study period.^8^

### Data extraction

Data extraction was performed individually and in an anonymized manner.^9^The definition of RA for the search in the administrative data systems was guided by constructing algorithms ( Web Supplemental Material, Section 3 ) in alignment with the International Disease Codes (ICDs-10) for RA and its subclassifications (ICD-10 from M05 to M08), in association with the codes of procedures of the Clinical Protocols and Therapeutic Guidelines (PCDT, acronym in Portuguese) for RA authorized and covered by SUS.^10^

Data were integrated from three DATASUS administrative data systems: High-Cost Procedure Authorization (APAC), Individualized Ambulatory Production Bulletin (BPAi), and Hospital Admission Authorization (AIH). For details of these systems, please check the Web Supplemental Material ( Figure S1 ). The APAC system includes information on date of birth, sex, state, city, ICD rheumatic disease code, treatment phase (initial or return), immunosuppressive treatment, dialysis (yes or no), and professional visits during the study period. The AIH system includes the dates of hospitalization, discharge and birth, sex, city, length of hospitalization, ICD reason for hospitalization, date and reason of death when applicable, type of hospitalization (urgency or emergency), and reason for dehospitalization (cure or death). The BPAi system includes patient data such as national health card number, sex, date of birth, race, color, and ethnicity, in addition to the code of the municipality of residence. It also includes the National Registry of Healthcare Establishments (CNES, acronym in Portuguese) number of the performing establishment, procedure code, quantity of procedures performed, date, and respective ICD. The scope of the extraction was national, carried out between 2008 and 2018.

### Eligibility criteria and case definition

Patients with RA were selected if they were over 18 years old, as described by the date of birth in the DATASUS forms used in the study. Patients had to have continuous information in at least one of the databases for up to 6 consecutive months prior to the baseline date. Patients with more than 20% missing information in at least two study variables were excluded ( Figure 1 ). The patient selection flowchart and the number of patients included in the study are provided in Figure 1 .

**Figure 1:** – Patient follow-up and selection flowchart. Notes: Patient follow-up flowchart shows the procedures to identify from the beginning to the end of data extraction. Patient selection flowchart shows the selection of patients included in the study, highlighting patients with a follow-up of 48 months and undergoing treatment with disease-modifying antirheumatic drugs (n = 4,231). AIH: Individualized Ambulatory Production Bulletin; APAC: Authorization for a High-Cost Procedure by the Brazilian Unified Health System; BPAi: Hospital Admission Authorization; CV: cardiovascular; RA: rheumatoid arthritis. Source: Prepared by the authors.

### Data cleaning and variable construction

After the initial data extraction, a thorough cleaning process was conducted to ensure data quality and consistency. This included removing duplicate entries, resolving inconsistencies, and addressing missing data. Variables were constructed based on the available data, ensuring they accurately reflected the patients’ demographic, clinical, and socioeconomic characteristics.

Initially, an exploratory analysis of the distribution of drugs described in the data systems was performed. Treatment information is specifically obtained in the APAC database, which concentrates information on the use of high-cost medications. Based on this distribution and the described assumptions, patients with RA were categorized as add-on, constant, or switch. Add-on patients are those who added any specific medication for RA during follow-up. Constant patients are those who did not change the therapeutic regimen during follow-up. Patients in the switch group changed the therapeutic regimen during follow-up. The constant group was subclassified as synthetic and biological. The switch group was subclassified according to the change from biological DMARD to synthetic DMARD or vice versa, or even when there was a change between drug classes of the same categorization, i.e., from one biological drug to another or from one synthetic drug to another ( Web Supplemental Material, Table S1 ). The following DMARDs were evaluated in the synthetic group: methotrexate, leflunomide, chloroquine, or hydroxychloroquine, sulfasalazine. The biological group included the following: adalimumab, infliximab, etanercept, certolizumab, golimumab, tocilizumab, abatacept, and rituximab.

### Profile of clinical comorbidities

For this study, RA severity was categorized according to the type of disease-specific treatment received during follow-up. The information regarding the treatment contained in the data systems was considered ( Web Supplemental Material, Table S2 ), based on the following principles: (1) in line with Brazilian guidelines used for this study, biological medication is provided as a second-line treatment, and its introduction suggests a failure of the first-line regimen;^10^(2) treatment changes reflect a change in health status, i.e., any addition or change of treatment would reflect clinical worsening, while the removal of a given medication from the information system was considered a proxy for improvement in the health status of the patient with RA.^11^

Socioeconomic information related to the place of residence was also obtained and analyzed, with the smallest geographic unit available for analysis being the municipality and the health district where the disease is treated. This survey was based on information from the IFDM, which is a synthetic indicator of municipal quality of life ( Web Supplemental Material, Section 4 ). The IFDM annually monitors the socioeconomic development of all Brazilian municipalities in 3 areas of activity: employment/income, education, and health.^12^This indicator is built based solely on official public statistics made available by the Ministries of Labor, Education, and Health in Brazil, and can be read in general aspects or analyzed by sector in terms of employment/income, health, and education. The index ranges from 0 (minimum) to 1 point (maximum) to classify the level of each location into the following 4 categories: low (0 to 0.4), regular (0.4 to 0.6), moderate (0.6 to 0.8), and high (0.8 to 1) ( Web Supplemental Material, Table S3 ).

### Cardiovascular risk factors

Cardiovascular risk factors were identified from all DATASUS administrative data systems based on the presence of the set of information grouped by the ICD-10 of the morbidity rate in association with the codes of procedures for their care. Cardiovascular events were defined as ACAD, heart failure, and CVA. Cardiovascular events were categorized by age group (> 40, 40 to 60, 60 to 70, 70 to 80, > 80 years). The profile of clinical comorbidities was defined with a focus on mapping traditional cardiovascular risk factors, including dyslipidemia, hypertension, diabetes, obesity, and smoking.

### Statistical analysis

The distribution of patients at baseline was described based on demographic data, RA data (categorized by treatment), traditional risk factors for cardiovascular events, and socioeconomic factors (IFDM). The categorical variables were described using absolute and relative frequencies. After this step, descriptive analysis of cardiovascular outcomes was performed. A logistic regression model was then used to explore baseline factors associated with the occurrence of cardiovascular outcomes in the studied population. The steps for building the model were as follows:

1. Sample pairing based on the presence of cardiovascular outcomes using variables such as sex, age group, region, diabetes, obesity, hypertension, smoking, and dyslipidemia, with a pairing ratio of 1:5.
2. Chi-square test to verify differences between treatment models given the presence of the outcome. ^13^
3. Paired post-hoc test to compare proportions and identify differences between models when the chi-square test was significant.
4. Bonferroni method to corroborate p-value results (the statistical significance adopted was p < 0.05). ^14^
5. Calculation of odds ratios to reveal statistically significant results.

The analysis places the use of the statistical tools in an appropriate order, with the chi-square test initially applied to detect differences between treatment models based on the presence of outcomes, which is suitable for independent samples. Secondly, the stepwise model was used to select which variables would be associated with outcomes. In this process, we first excluded the variable model of treatment. In sequence, the variables were selected by the stepwise model as criteria in pairing among patients with outcomes and patients without outcomes. With the paired patients, we used the test of proportions to verify in which subgroup inside the treatment model there was a statistical significance. This was done to be sure that the effect of other variables would be nulled and to exclude confusion effect in the magnitude of treatment effect in the group of patients with outcomes. After verifying in which group there was a difference, we calculated its corresponding odd ratio and p value, considering p < 0.001 ( Web Supplemental Material, Table S8 ). This approach allowed us to control for confounding variables and conduct an accurate analysis of subgroups.

The study was submitted and approved by the Institutional Research Ethics Committee, under opinion number 23016819.9.0000.5257. Data extraction was provided by DATASUS management through process number SEI 250001348282015-86.

## Results

### Study population

Initially, a total of 37,941 patients with a diagnosis of RA were identified, of whom 7,225 were followed up for a period of 48 months, and 4,231 of them were treated with DMARDs. The study’s analysis, therefore, included 4,231 patients with RA treated with DMARDs and followed for 48 months ( Figure 1 ). The majority of the patients were female (80.3%), with a mean age of 54.2 years. Most patients lived in the Southeast Region (57.2%), and 47.5% had IFDM above 0.8. Table 1 presents social, demographic, and clinical distribution of the treated population in relation to baseline and 48 months. The Central Illustration provides an overview of the social, demographic, and clinical distribution of the population with RA and cardiovascular outcomes.

**Table 1:** – Social, demographic, and clinical distribution of the treated population (baseline and 48 months)

Variable	Baseline (n = 15,999)	48 Months (n = 4,231)	Observations
Sex			
Female	12,779 (79.9%)	3,398 (80.3%)	Predominantly female patients.
Male	3,220 (20.1%)	833 (19.7%)	
Age			
Average	50.08 (13.82)	54.22 (12.83)	Increase in average age over 48 months.
< 40 years	3,888 (24.3%)	648 (15.3%)	Decrease in younger patients.
40 to 60 years	8,480 (53.0%)	2,210 (52.2%)	Consistent proportion of middle-aged patients.
60 to 70 years	2,645 (16.5%)	993 (23.5%)	Increase in older patients.
70 to 80 years	862 (5.4%)	324 (7.7%)	
> 80 years	124 (0.8%)	56 (1.3%)	
Race			
White	7,559 (47.2%)	2,138 (50.5%)	Predominantly white.
African Descent	3,440 (21.5%)	834 (19.7%)	
Asian	312 (2.0%)	84 (2.0%)	
Region			
Midwest	898 (5.6%)	195 (4.6%)	
North	551 (3.4%)	91 (2.2%)	
Northeast	2,332 (14.6%)	474 (11.2%)	
South	3,268 (20.4%)	1,050 (24.8%)	
Southeast	8,950 (55.9%)	2,421 (57.2%)	Predominantly from the Southeast Region.
IFDM			
> 0.8	7,086 (44.3%)	2,010 (47.5%)	Majority with high IFDM.
IFDM employment/income			
0.6 to 0.7	7,036 (44.0%)	1,756 (41.5%)	
IFDM health			
> 0.8	12,492 (78.1%)	3,443 (81.4%)	Majority with high IFDM health.
IFDM education			
> 0.8	11,251 (70.3%)	3,031 (71.6%)	Majority with high IFDM education.
CV risk factors			
0	15,485 (96.8%)	4,102 (97.0%)	Majority with no risk factors (p = 0.003).
≥ 1	514 (3.2%)	129 (3.0%)	

This table shows a comprehensive demographic and clinical profile of the patients with rheumatoid arthritis at baseline and after 48 months. There is a noticeable increase in the average age and the proportion of older patients over the study period. The majority of patients are from the Southeast Region and have a high IFDM in the health and education sectors. CV: cardiovascular; IFDM: FIRJAM Index for Human Development. Source: Prepared by the authors.

### Distribution and categorization of DMARDs

Biological DMARDs were used by 1,885 (44.6%) patients, while synthetic DMARDs were used by 3,757 (88.8%) patients. Patients were categorized based on their treatment trajectories as follows: constant synthetic (20.4%), synthetic switch (35.1%), constant biological (7.9%), add-on medication (20.2%), synthetic-to-biological switch (10.5%), biological switch (3.3%), and biological-to-synthetic switch (2.6%). The synthetic switch group showed a higher prevalence of traditional cardiovascular risk factors ( Web Supplemental Material, Table S4 ).

### Cardiovascular outcomes

During the follow-up period, 198 cardiovascular outcomes were recorded in 184 patients, including heart failure, acute coronary syndromes, and stroke. As illustrated in Figures 2 and 3 , most outcomes occurred in the switch group (130 [65.7%]), followed by the constant group (42 [21.2%]) and the add-on group (26 [13.1%]). Outcomes were most frequent in the synthetic switch group (112 [56.6%]), followed by the constant synthetic group (33 [16.6%]), the add-on group (26 [13.3%]), the synthetic-to-biological switch group (12 [6%]), the constant biological group (9 [4.5%]), the biological switch group (3 [1.5%]), and the biological-to-synthetic switch group (3 [1.5%]) ( Web Supplemental Material, Table S5 ).

**Figure 2:** – Distribution of cardiovascular outcomes in relation to grouped categories of treatment. Description: Bar chart showing the distribution of cardiovascular outcomes by treatment categories. Add-on: addition of a new biological or synthetic medication; constant: the same drug during the study; switch: change from one drug to another during the study. ACAD: acute coronary artery disease; CVA: cerebrovascular accident. Source: Prepared by the authors.

**Figure 3:** – Distribution of cardiovascular outcomes in relation to each category of treatment for rheumatoid arthritis. Description: Detailed chart showing the distribution of cardiovascular outcomes by specific treatment categories. Add-on: addition of biologic or synthetic; ACAD: acute coronary artery disease; BC: biological constant; BS: biological switch; BSS: biological-to-synthetic switch; CVA: cerebrovascular accident; SBS: synthetic-to-biological switch; SC: synthetic constant; SS: synthetic switch. Source: Prepared by the authors.

### Characteristics of patients with cardiovascular outcomes

The distribution of cardiovascular outcomes at the end of the 48-month follow-up period was evaluated in relation to the total of 198 cardiovascular outcomes. The cardiovascular outcomes identified were: ACAD (ICD-10 I20-I24) with 91 events (46%), heart failure (ICD-10 I50) with 80 events (40.4%), and CVA (ICD-10 I64/G45) with 27 events (13.6%) ( Figure 3 ). Of the 198 cardiovascular outcomes, 77.2% occurred in females; 41.3% of patients were aged 40 to 60 years, with a mean age of 59.93 years. Most patients were white (66.8%) and lived in the Southeast Region (56.5%). The majority had an overall IFDM greater than 0.8 (52.7%), particularly in the health (83.2%) and education (71.7%) domains, while the income/employment domain ranged from 0.6 to 0.7 (40.8%). We observed that the IFDM income/employment domain was predominantly between 0.6 and 0.7 (40.8%) in the group with cardiovascular outcomes, as well as for the general group (41.5%), although it is worth noting that IFDM was analyzed across all Brazilian regions, and no statistical significance was observed (p > 0.05) ( Web Supplemental Material, Table S6 ).

### Association with traditional cardiovascular risk factors and outcomes

Cardiovascular outcomes were more common in patients with traditional risk factors, with a direct association between the presence of one or more risk factors and cardiovascular outcomes (odds ratio of 2.39; p = 0.003) ( Figure 4 ). There was an increasing relationship between age and the occurrence of cardiovascular outcomes, with higher odds ratios for older age ranges. Patients in the synthetic switch group had a higher likelihood of cardiovascular outcomes compared to the constant synthetic group (odds ratio of 2.31; p = 0.002) ( Table 3 ; Web Supplemental Material, Table 7A and 7B ).

**Figure 4:** – Logistic regression model for cardiovascular outcomes. Description: Graphical representation of the logistic regression model showing the odds ratios for cardiovascular events. OR: odds ratio for cardiovascular events. Source: Prepared by the authors.

**Table 3:** – Distribution of DMARDs used in REAL study and this study, pointing to cardiovascular outcomes in this study, according to which DMARDs patients received

	Type of DMARDs	REAL Study and DMARDs	This study and DMARDs	Cardiovascular outcomes
Patients	-	N = 1,116	N = 4,231	N = 184
Methotrexate	Synthetic	66.5%	31.9%	29.9%
Leflunomid	Synthetic	33.9%	59.6%	60.9%
Chloroquine or Hydroxychloroquine	Synthetic	13.1%	18.4%	17.4%
Sulfasalazine	Synthetic	4.9%	11.3%	15.2%
Anti-TNF (adalimumab, infliximab, etanercept, certolizumab, golimumab)	Biological	19.9%	53.4%	31.0%
Tocilizumab	Biological	4.9%	1.7%	0
Abatacept	Biological	6.5%	2.0%	0
Rituximab	Biological	4.4%	1.5%	1.1%
Synthetic group	-	-	3,757 (88.8%)	172 (93.5%)
Biological group	-	-	1,885 (44.6%)	51 (27.7%)

The table specifies the association of cardiovascular outcomes and synthetic and biological DMARDs used in the REAL study and in our study. DMARDs: disease-modifying antirheumatic drugs. Source: Prepared by the authors.

## Discussion

The increased risk of cardiovascular events in RA is not fully explained by traditional risk factors alone.^2^This retrospective cohort study aimed to identify both traditional and non-traditional risk factors associated with cardiovascular outcomes in patients with RA registered in DATASUS ( Web Supplemental Material, Table S9 ).

### Findings on cardiovascular events

We identified 198 cardiovascular events within our cohort. Aging was a prevalent factor among patients with cardiovascular events, with hypertension being the most common traditional risk factor associated with these events. Although the proportion of traditional risk factors found in our study was relatively low compared to other studies, our findings align with other primary data collection studies conducted in Brazil. For instance, the reported prevalence of hypertension in patients with RA varies significantly, ranging from 4% to 73%, depending on the study design and population.^15 , 16^Simard et al.^17^showed the prevalence of diabetes in RA to be between 7.0% and 35.0%, with few studies indicating a higher prevalence of diabetes in RA^18^( Web Supplemental Material, Table S10 ).

### Impact of DMARDs on cardiovascular risk

Vicente et al.^19^conducted a cross-sectional analysis of patients from the REAL study cohort at baseline, finding that cardiovascular risk is high in the population with RA. Their study suggested that the choice of DMARDs, particularly biological DMARDs, could result in better cardiovascular outcomes compared to synthetic DMARDs ( Table 3 ). Our cohort study corroborated these findings, showing an association between the type of RA treatment and the occurrence of cardiovascular outcomes.^20^Patients who needed to change their medication to control disease activity (switch group) were more likely to develop cardiovascular outcomes. Golmia et al.^21^presented a direct comparison of the use of infliximab and tocilizumab, two biological DMARDs, and verified different responses between them concerning RA functionality.

### Statistical model and key associations

The logistic regression model in our study verified that age progression and the presence of one or more cardiovascular risk factors were significantly associated with cardiovascular outcomes. The model showed a direct association between the presence of traditional cardiovascular risk factors and cardiovascular outcomes, with an odds ratio of 2.39 (1.34 to 4.29; p = 0.003) ( Table 2A , Figure 4 ). In our paired analysis, we observed a significant difference in cardiovascular outcomes among patients who had to switch their synthetic drugs, suggesting that these patients might have more severe disease ( Table S8 ).

**Table 2:** – Regression model for different variables and association with cardiovascular outcomes and test result comparing the proportions between types of treatment

Variable	OR (95% CI)	p value (Wald's Test)		Note
Table 2A Age (reference: < 40 years)				
40 to 60 years	1.48 (0.85 - 2.60)	0.169		No significant association
60 to 70 years	2.56 (1.44 - 4.57)	0.001		Significant association
70 to 80 years	3.53 (1.84 - 6.78)	<0.001		Significant association
> 80 years	6.60 (2.65 - 16.43)	<0.001		Significant association
Risk factor (reference: 0)				
≥ 1 versus 0	2.39 (1.34 - 4.29)	0.003		Significant association
**Comparison**	**OR (95% CI)**	**p value**	**Note**	
Table 2B				
Switch versus constant	1.81 (1.23 - 2.71)	0.056	Close to significance	
Table 2C				
Synthetic switch versus constant synthetic	2.31 (1.50 - 3.66)	0.002	Significant association	
Synthetic switch versus add-on	1.81 (1.15 - 2.96)	0.16	Not significant	

Table 2A highlights the increasing odds of cardiovascular outcomes with advancing age. The presence of one or more risk factors significantly increases the likelihood of cardiovascular events. Table 2B and 2C shows a significant association between synthetic switch treatment and cardiovascular outcomes compared to constant synthetic treatment. CI: confidence interval; OR: odds ratio. Source: Prepared by the authors.

### Role of socioeconomic factors

In addition to corroborating existing literature, our study advances the discussion of disparities and the role of access as a risk factor for cardiovascular events.^22^The socioeconomic index (IFDM) in our study did not show a statistically significant correlation with cardiovascular outcomes in the global RA population (p > 0.05). This contrasts with other studies that indicate 80% of cardiovascular risk in RA arises in countries with low to medium socioeconomic status,^23^but less than 20% of clinical studies include socioeconomic status in their models.^20^

### Strengths and limitation

This study has several strengths, including a large, nationwide sample size and a comprehensive analysis of both clinical and socioeconomic factors, providing valuable insights into the cardiovascular risks faced by patients with RA in Brazil. The use of the DATASUS database allowed for the inclusion of a diverse patient population across various healthcare settings. However, the study focuses on patients receiving high-complexity medications for severe RA, which may not represent those with early or mild-to-moderate disease. Some limitations include potential variations in data accuracy, unaccounted residual confounding factors (such as diet, physical activity, genetic predispositions, medication adherence), and the absence of detailed clinical data (for example, disease activity scores and laboratory results). Changes in clinical practices and healthcare quality over the study period could also have influenced the results. Despite these limitations, the strengths of this study help to mitigate these concerns. Future research could focus on patients with early-stage RA or those with mild-to-moderate disease severity to further expand these findings.

### Implications for future research and policy

The relevance of this study lies in its exploration of cardiovascular aspects in a large nationwide sample of patients with RA in Brazil. Our findings reinforce the premise that better inflammation control translates into reduced cardiovascular outcomes in patients with RA. As a future perspective, this evidence can guide comparative effectiveness research with the therapeutic options analyzed here, using cardiovascular events as outcome measures. This approach can also include cost-comparative studies, which may be useful for designing public policies both in Brazil and in the context of the global burden of disease.

Furthermore, our study’s findings can inform healthcare providers and policymakers about the importance of tailored treatments for patients with RA, considering both clinical and socioeconomic factors to optimize cardiovascular health outcomes.

## Conclusion

Our study demonstrates that traditional cardiovascular risk factors, socioeconomic factors, and inflammation-related factors significantly impact cardiovascular outcomes in patients with severe RA receiving high-complexity biological treatments. The findings highlight the importance of comprehensive cardiovascular risk management and the benefits of inflammation control through appropriate DMARDs, particularly biological DMARDs. While the study’s large, nationwide sample and detailed analysis provide valuable insights, future research should focus on patients with early-stage or mild-to-moderate RA to further understand cardiovascular risks across different disease stages and consider residual confounding factors and detailed clinical data to enhance the understanding of these relationships.
